# Breast cancer-specific mortality in early breast cancer as defined by high-risk clinical and pathologic characteristics

**DOI:** 10.1371/journal.pone.0264637

**Published:** 2022-02-25

**Authors:** David R. Nelson, Jacqueline Brown, Aki Morikawa, Michael Method

**Affiliations:** 1 Eli Lilly and Company, Indianapolis, IN, United States of America; 2 Eli Lilly and Company, Bracknell, Surrey, United Kingdom; 3 Division of Hematology/Oncology, Department of Internal Medicine, University of Michigan, Ann Arbor, MI, United States of America; Brown University Warren Alpert Medical School, UNITED STATES

## Abstract

**Objectives:**

To investigate breast cancer-specific mortality by early breast cancer (EBC; Stages I-IIIC) subtype; incidence of high-risk indicators for recurrence (defined in monarchE trial); and mortality risk difference by those who did/did not meet these criteria.

**Materials and methods:**

Analyses included patients with initial EBC diagnosis between 2010–2015 from Surveillance, Epidemiology, and End Results (SEER) data (n = 342,149). Cox proportional hazards models and Kaplan-Meier estimates examined mortality among 228,031 patients, by subtype (hormone receptor [HR]-positive [+], human epidermal growth factor receptor-2 [HER2] negative [–]; triple negative [TNBC]; HR+, HER2+; HR-, HER2+). Incidence and mortality among patients who did/did not meet monarchE clinicopathological high-risk criteria were examined.

**Results:**

Among patients with HR+, HER2- EBC, histologic Grade 3 (vs. Grade 1) was the most influential factor on mortality (hazard ratio, 3.61; 95%CI, 3.27, 3.98). Among patients with TNBC, ≥4 ipsilateral axillary positive nodes (vs. node negative) was the most influential factor on mortality (hazard ratio, 3.46; 95%CI, 2.87, 4.17). For patients with HR-, HER2+ or HR+, HER2+ EBC, tumor size ≥5 cm (vs. <1 cm) and ≥4 ipsilateral axillary positive nodes were the most influential factors on mortality. The 60-month mortality rate for the 12% of patients within the HR+, HER2- EBC group meeting monarchE clinicopathological high-risk criteria was 16.5%, versus 7.0% (Stage II–III and node positive) and 2.8% (Stage I or node negative) for those not meeting criteria. The 60-month mortality rate for patients with TNBC was 18.5%.

**Conclusion:**

Mortality risk and the relative importance of risk factors varied by subtype. monarchE clinicopathological high-risk criteria were associated with increased mortality risk among patients with HR+, HER2- EBC. Patients with HR+, HER2- EBC, and monarchE clinicopathological high-risk criteria experienced risk of mortality similar to patients with early TNBC. These data highlight a high unmet need in this select patient population who may benefit most from therapy escalation.

## Introduction

Breast cancer is the most commonly diagnosed cancer and the second leading cause of cancer deaths among women in the United States (US) [1]. A US population-based study showed the most common breast cancer subtype is hormone receptor (HR)-positive (+), human epidermal growth factor receptor 2 (HER2)-negative (-), occurring in 73% of all patients, followed by triple negative (HR-, HER2- [TNBC]; 12%), HR+, HER2+ (10%), and HR-, HER2+ (5%) [2].

Early breast cancer (EBC; Stages I, II, or III) accounts for >90% of all diagnosed breast cancers [2]. Despite the availability of EBC treatment options with curative intent, including primary surgery, radiation, chemotherapy, and adjuvant endocrine therapy (ET), nearly 30% of patients diagnosed with EBC will experience breast cancer recurrence [3], many with distant metastases [4], which is incurable. Studies have shown a subset of patients with high-risk clinical features (ie, large primary tumor size, more advanced Stage, greater extent of axillary lymph node (ALN) involvement, and high histologic Grade) are at a higher risk of recurrence [5–7]. In HR+, HER2- breast cancer, highly proliferative disease, as demonstrated by Ki-67 index ≥20% and several multi-gene assays, has also been shown to be associated with higher risk of disease recurrence [5, 8–12]. Identifying patients with a high risk of recurrence will help optimize treatment, while potentially avoiding overtreatment in patients who are less likely to benefit [13, 14].

Surgery, radiotherapy, adjuvant/neoadjuvant chemotherapy, and ET are all considered standard treatment options for patients with HR+, HER2- EBC and vary according to recurrence risk [15, 16]. Although ET been an established treatment for HR+, HER2- EBC, there has been little advancement over the past two decades, and it remains that up to 20% of patients will experience recurrence in the first 10 years [17]. The 5-year survival rate for patients with distant metastases at diagnosis in the US is just 28% [18].

Recently, results were published from monarchE (I3Y-MC-JPCF), a randomized Phase III, open-label trial that investigated whether the addition of abemaciclib, a cyclin-dependent kinase 4 and 6 dual inhibitor, to ET in the adjuvant setting provided additional benefit for patients with HR+, HER2- EBC [19]. This trial enrolled 5637 patients, who were considered at high risk of early recurrence [19, 20]. In monarchE, among patients with HR+, HER2- invasive EBC, high risk of recurrence was defined as having either ≥4 positive axillary lymph nodes (ALNs), or 1–3 positive ALNs in combination with ≥1 of the following high-risk features: primary invasive tumor size ≥5 cm, histological Grade 3 tumor, or tumor with central Ki-67 index ≥20% [19, 20]. The pre-planned interim and primary outcome analyses demonstrated that, compared with ET alone, the addition of abemaciclib to ET was associated with decreased risk of recurrence or death [19, 20]. Improvements in both invasive disease-free survival (IDFS) and distant relapse-free survival (DRFS) were noted [19, 20]. Based on the results from this clinical trial, the Food and Drug Administration (FDA) approved Verzenio (abemaciclib) in combination with ET (tamoxifen or an aromatase inhibitor) for the adjuvant treatment of adult patients with HR+ HER2-, node-positive EBC at high risk of recurrence and a Ki-67 score of ≥20% as determined by an FDA-approved test [21]. The use of abemaciclib in adjuvant high-risk EBC has been endorsed in the American Society of Clinical Oncology (ASCO) guidelines (15 November 2021) [22] and National Comprehensive Cancer Network (NCCN) guidelines (23 November 2021) [23].

Real-world data on the relative contribution of clinical and pathologic characteristics to breast cancer-specific mortality, by breast cancer subtype according to HR and HER2 status, as well as the incidence of patients who meet the monarchE clinicopathologic high-risk criteria and their associated survival outcomes relative to those who do not fulfill the criteria, will assist in identifying those patients with EBC who stand to benefit most from therapy escalation.

The objectives of this study were to examine and identify differences in breast cancer-specific mortality risk factors of interest among patients with EBC in the Surveillance, Epidemiology, and End Results (SEER) registry, by HR, HER2 subtype, and to evaluate the relative contribution of those clinical and pathologic characteristics of interest to breast cancer-specific mortality by subtype. Additional objectives were to describe the incidence of monarchE clinicopathological high-risk criteria (ie, without the Ki-67 index ≥20% tumor eligibility criterion, as these data are not available in SEER) [19, 20] within the HR+, HER2- EBC population in SEER, and to determine if breast cancer-specific mortality risk differed by those patients who did and did not meet monarchE clinicopathological criteria for high risk of recurrence. The goal of these additional objectives was to quantify and contextualize the difference in prognosis within the HR+, HER2- subtype.

## Materials and methods

### Data source

**Fig 1:**
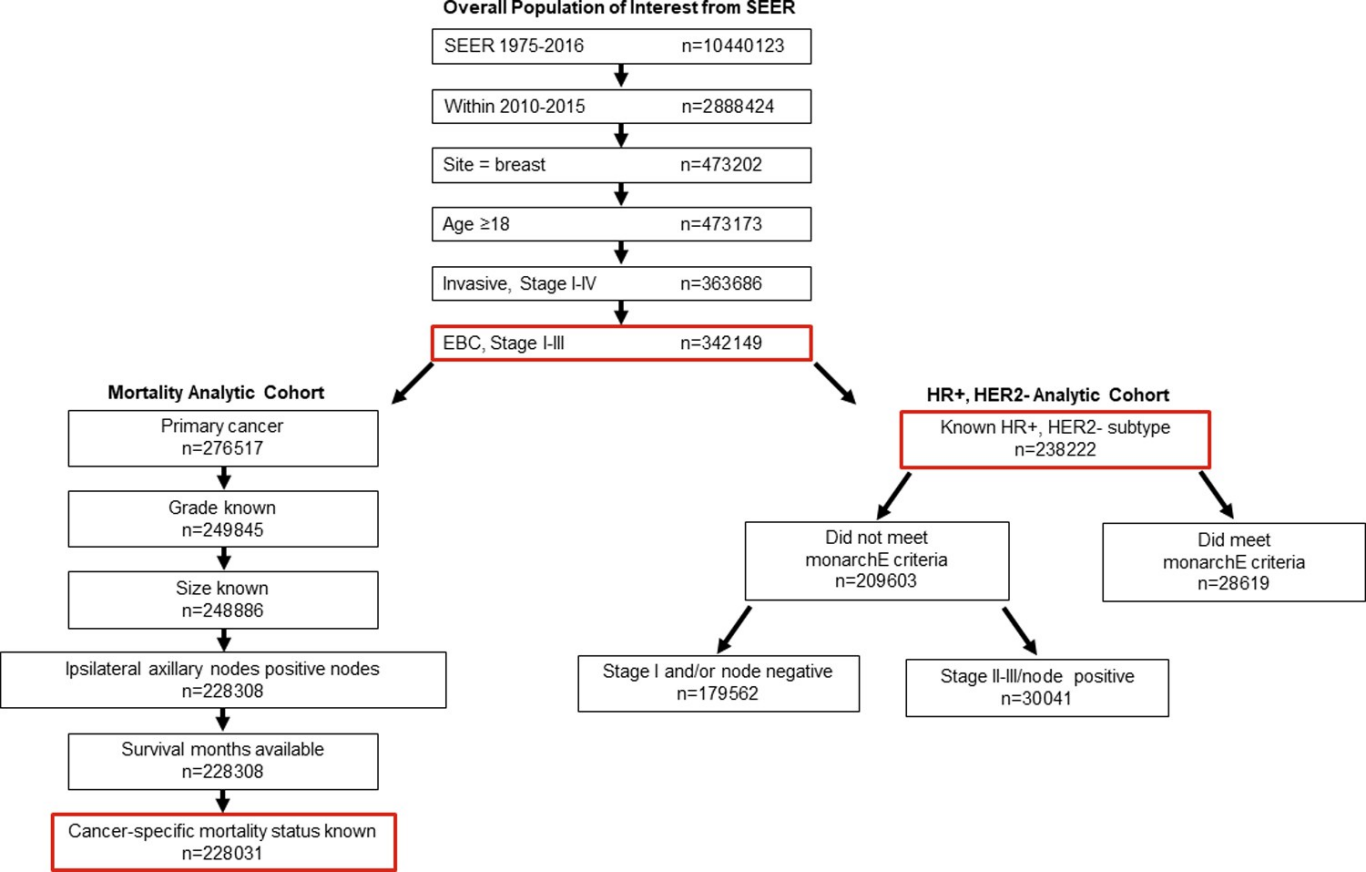
Patient attrition from the overall SEER population based on defined inclusion and exclusion criteria. Abbreviations: EBC, early breast cancer; HER, human epidermal growth factor receptor; HR, hormone receptor; SEER, Surveillance, Epidemiology, and End Results.

SEER collects and publishes cancer incidence and survival data from population-based cancer registries, covering approximately 34% of the total US population in 19 geographic area to include demographic and cancer-based clinical and mortality information [24]. The SEER Registries Research Data, November 2018 submission (1975–2016) database [25] was used to identify eligible patients. There were 342,149 patients in SEER who met initial inclusion criteria: initial diagnosis between 2010–2015, cancer site being the breast, age ≥18, and Stage I-III EBC (derived from the *American Joint Committee on Cancer*, *[AJCC]*, *Cancer Staging Manual* 7^th^ edition) (**Fig 1**).

The primary outcome of this study was breast cancer-specific mortality (described from here forward as mortality). Survival time was defined as time (in months) from initial diagnosis to time of death, and patients censored for death were followed until the last recorded visit date in SEER. Subtype was defined in this study by joint HR, HER2 status, and included: HR+, HER2-; TNBC (HR-, HER2-); HR+, HER2+; HR-, HER2+; and unknown subtypes. Mortality analyses were conducted among a subset of patients (referred to as mortality analytic cohort; **Fig 1**) who had the additional inclusion criteria of this being the primary cancer and having complete data on key variables of interest (histologic Grade, tumor size, ipsilateral axillary nodes positive, survival months available, and cancer-specific mortality status known). Histologic Grade was defined based on collaborative Stage Site-Specific Factor 7, per the Nottingham or Bloom-Richardson Score/Grade. Tumor size was defined based on collaborative Stage tumor size. Ipsilateral axillary nodal positivity was defined per the *AJCC Cancer Staging Manual*, 7^th^ edition, and included micrometastases (pN1mi) and macrometastases. Micrometastases were defined as tumor deposits larger than 0.2 mm but not larger than 2.0 mm in the largest dimension. Macrometastases were defined as having a tumor deposit in at least 1 node larger than 2 mm. For the purposes of this analysis, ALN involvement was analyzed in the following groups: node negative; having 1–3 positive micrometastases in ALNs; having ≥4 positive micrometastases in ALNs (both representing pN1mi as defined); having 1–3 positive ALNs (ie, macrometastases, pN1); or having ≥4 positive ALNs (ie, macrometastases, pN2/3). Of note, creating separate analytic groups for 1–3 positive micrometastases and ≥4 positive micrometastases was done, despite both subgroups being classified in AJCC staging criteria as pN1mi, to evaluate the potential impact of multiple micrometastases on mortality. A detailed list of all risk factors of interest extracted from the ASCII text version of SEER data [26] is included in the (**S1 Table**).

A separate subset of patients with known HR+, HER2- subtype (referred to as HR+, HER2- analytic cohort) was identified to examine mortality among patients who did and did not meet monarchE high-risk criteria (**Fig 1**).

This observational study used de-identified and publicly available data from SEER and thus did not require formal consent or institutional review board approval. This study was conducted in accordance with the Declaration of Helsinki.

### Statistical analysis

#### Breast cancer-specific mortality

Within the mortality analytic cohort, survival curves by HR, HER2 subtype were estimated using Kaplan-Meier methods with 95% Hall-Wellner confidence bands [27] and compared with log-rank tests. Multivariable Cox proportional hazards regression models assessed mortality risk factors (subtype, age, sex, race/ethnicity, disease Stage, nodal status, histologic Grade, tumor size, histology) across the total cohort and by subtype. Among the largest subtype (HR+, HER2-), these models were repeated by disease Stage, so risk factors could be assessed independent of Stage. Only the HR+, HER2- subtype allowed analyses at the Stage level of granularity due to small sample sizes for other subtypes.

Chi-squared statistics estimated from the multivariable Cox proportional hazards regression models were used to examine the relative importance of risk factors within each subtype. Chi-square values generated within Cox models are sensitive to the sample size, with the chi-square value increasing with increasing sample size. In this analysis, to adjust for differences in the sample size of the subtypes, the chi-square values were divided by subtype sample size [28]. Applying this method quantified the relative importance of each factor in the subtype-specific model, combining the effect size and the incidence of the risk factors. Details regarding adjusting for multiplicity are included in the (**S1 Text**) The mortality analytic cohort was compared to the group of patients excluded due to lack of mortality data using chi-square tests.

#### monarchE comparisons

In SEER, results on Ki-67 immunohistochemistry testing were not available, so the high-risk definition was based only on the monarchE high-risk clinicopathologic criteria, corresponding to the criteria used for the 91% of patients (n = 5120 of n = 5637) in the monarchE trial and referred to as Cohort 1 [20]: HR+, HER2- invasive EBC with either ≥4 positive ALNs, or 1–3 positive ALNs in combination with ≥1 of the following high-risk features: primary invasive tumor size ≥5 cm or histological Grade 3 tumor.

Within the HR+, HER2- analytic cohort, descriptive statistics were used to detail the percentage of patients with HR+, HER2- EBC who did and did not meet the above defined high-risk criteria. Among patients who did not meet the high-risk criteria, to reflect different levels of risk, we also examined the difference between patients who had EBC that was Stage II-III and node positive but did not meet the monarchE clinicopathological high-risk criteria versus those who did not meet the monarchE clinicopathological high-risk criteria due to having EBC that was Stage I and/or node negative. Cox proportional hazard models (adjusted for demographics: age, sex, and race/ethnicity), and Kaplan-Meier methods with log-rank tests compared survival for those who did and did not meet the monarchE clinicopathological high-risk criteria.

#### Software

All analyses utilized SAS software version 9.4 (SAS Institute Inc., Cary, NC, US).

## Results

### Demographic and clinical characteristics

Attrition from the overall SEER database is described in **Fig 1**. As described above, 342,149 patients met initial inclusion. Patients were mostly female (99.3%), had a mean age of 61.7 years (standard deviation, 13.5), and were predominantly non-Hispanic White (69.1%). Consistent with prior epidemiologic studies in the US, the most common breast cancer subtype within this cohort was HR+, HER2- (69.6%), followed by TNBC (10.5%), HR+, HER2+ (9.6%), and HR-, HER2+ (4.0%); there were 21,788 (6.4%) patients with EBC, and unknown HR, HER2 status (**S1 Fig**). There was variation in the representation of non-Hispanic Black patients, with a higher percentage of non-Hispanic Black patients within the TNBC (19.8%), compared with other subtypes (**Table 1**).

**Table 1:** Demographic and clinical characteristics among 342,149 patients with EBC, by HR/HER2 subtype.

Demographic or clinical characteristic	HR+, HER2-		TNBC		HR+, HER2+		HR-, HER2+		Other^a^	
	n = 238 222		n = 35 761		n = 32 682		n = 13 696		n = 21 788	
	Count	%^b^	Count	%^b^	Count	%^b^	Count	%^b^	Count	%^b^
**Sex**										
Male	2 041	0.9	33	0.1	255	0.8	16	0.1	215	1.0
Female	236 181	99.1	35 728	99.9	32 427	99.2	13 680	99.9	21 573	99.0
**Age, mean** ^b^	58.2	13.7	58.3	13.2	62.7	13.2	59.0	14.0	63.1	14.2
**Age group**										
18–29	774	0.3	381	1.1	341	1.0	112	0.8	111	0.5
30–39	7 263	3.1	2 592	7.3	2 351	7.2	915	6.7	752	3.5
40–49	34 263	14.4	6 443	18.0	6 411	19.6	2 440	17.8	3 056	14.0
50–59	54 091	22.7	9 245	25.9	8 889	27.2	4 182	30.5	5 024	23.1
60–69	67 726	28.4	8 763	24.5	7 911	24.2	3 344	24.4	5 751	26.4
70–79	47 726	20.0	5 321	14.9	4 414	13.5	1 781	13.0	3 984	18.3
80–89	22 985	9.7	2 571	7.2	2 013	6.2	777	5.7	2 480	11.4
90+	3 394	1.4	445	1.2	352	1.1	145	1.1	630	2.9
**Race/ethnicity**										
Spanish-Hispanic-Latino	24 273	10.2	4 292	12.0	4 067	12.4	1 836	13.4	2 769	12.7
Non-Hispanic White	170 866	71.7	21 631	60.5	21 362	65.4	8 169	59.7	14 381	66.0
Non-Hispanic Black	21 247	8.9	7 083	19.8	3 678	11.3	1 868	13.6	2 443	11.2
Non-Hispanic American Indian/ Alaska Native	1 254	0.5	195	0.6	221	0.7	96	0.7	125	0.6
Non-Hispanic Asian or Pacific Islander	19 541	8.2	2 442	6.8	3 209	9.8	1 673	12.2	1 851	8.5
Non-Hispanic unknown	1 041	0.4	118	0.3	145	0.4	54	0.4	219	1.0
**Breast subtype**										
HR+, HER2-	238 222	100.0	-	-	-	-	-	-	-	-
TNBC	-	-	35 761	100.0	-	-	-	-	-	-
HR+, HER2+	-	-	-	-	32 682	100.0	-	-	-	-
HR-, HER2+	-	-	-	-	-	-	13 696	100.0	-	-
HR+, HER2 unknown	-	-	-	-	-	-	-	-	12 206	56.2
HR unknown, HER2 unknown	-	-	-	-	-	-	-	-	9 582	44.0
**Stage**										
Stage I NOS/IA	132 575	55.7	13 424	37.5	13 354	40.9	4 907	35.8	12 252	56.2
Stage IB	6 273	2.6	432	1.2	631	1.9	221	1.6	412	1.9
Stage IIA	50 950	21.4	11 046	30.9	8 405	25.7	3 462	25.3	4 366	20.4
Stage IIB	24 912	10.5	5 154	14.4	5 026	15.4	2 154	15.7	2 350	10.8
Stage III NOS/IIIA	14 188	6.0	2 860	8.0	2 939	9.0	1 411	10.3	1 244	5.7
Stage IIIB-C	9 324	3.9	2 845	8.0	2 327	7.1	1 541	11.3	1 164	5.3
**Nodal status**										
Node negative	173 289	72.7	24 121	67.5	20 350	62.3	7 976	58.2	16 731	76.8
Micrometastasis 1–3 positive ipsilateral axillary nodes	10 524	4.4	987	2.8	1 298	4.0	405	3.0	729	3.4
Micrometastasis ≥4 positive ipsilateral axillary nodes^c^	259	0.1	45	0.1	45	0.1	21	0.2	36	0.2
Node positive 1–3 positive ipsilateral axillary nodes^d^	36 764	15.4	6 759	18.9	7 275	22.3	3 326	24.3	2 685	12.2
Node positive ≥4 positive ipsilateral axillary nodes^d^	14 895	6.3	2 712	7.6	2 796	8.6	1 325	9.7	1 075	4.9
Unknown nodal status	2 491	1.1	1 137	3.2	918	2.8	643	4.7	532	2.4
**Histologic grade**										
Grade 1	73 826	31.0	1 506	4.2	2 739	8.4	516	3.8	4 265	19.6
Grade 2	108 164	45.4	5 959	16.7	12 531	38.3	2 996	21.9	6 644	30.5
Grade 3	38 567	16.2	24 440	68.3	13 859	42.4	8 194	59.8	4 366	20.0
Grade unknown	17 665	7.4	3 856	10.8	3 553	10.9	1 990	14.5	6 513	29.9
**Tumor size**										
<1 cm	57 934	24.3	4 857	13.6	5 744	17.6	2 754	20.1	7 315	33.6
≥1 cm to <2 cm	91 155	38.3	10 239	28.6	10 075	30.8	3 251	23.7	5 906	27.1
≥2 cm to <3 cm	44 962	18.9	8 590	24.0	7 432	22.7	2 874	21.0	3 588	16.5
≥3 cm to <4 cm	18 777	7.9	4 955	13.9	3 922	12.0	1 765	12.9	1 771	8.1
≥4 cm to <5 cm	9 443	4.0	2 581	7.2	2 061	6.3	1 018	7.4	983	4.5
≥5 cm	15 049	6.3	4 177	11.7	3 193	9.8	1 782	13.0	1 774	8.1
Size unknown	902	0.4	362	1.0	255	0.8	252	1.8	451	2.1
**Laterality**										
Left	117 898	49.5	17 400	48.7	15 973	48.9	6 664	48.7	10 557	48.5
Right	120 269	50.5	18 344	51.3	16 694	51.1	7 025	51.3	11 175	51.3
Other	55	0.02	17	0.1	15	0.1	7	0.1	56	0.3
**Histology**										
Other adenocarcinomas	2 338	1.00	838	2.3	387	1.2	250	1.8	357	1.6
Mucinous adenocarcinoma	6 019	2.5	37	0.1	315	1.0	38	0.3	400	1.8
Infiltrating duct carcinoma	169 355	71.1	30 251	84.6	27 643	84.6	12 118	88.5	15 208	69.8
Lobular carcinoma, NOS	28 272	11.9	434	1.2	1 318	4.0	135	1.00	1 711	7.9
Infiltrating duct mixed/infiltrating lobular mixed	26 251	11.0	1 535	4.3	2 244	6.9	514	3.8	1 572	7.2
Other^e^	5 987	0.03	2 666	0.1	775	0.02	641	0.1	2 540	0.1

^a^Other indicates HR+/HER2 unknown and HR/HER2 unknown.

^b^Where age is presented as a continuous variable, the value presented in this column is standard deviation rather than percentage.

^c^Per the *American Joint Committee on Cancer Staging Manual*, micrometastases were defined as tumor deposits larger than 0.2 mm but not larger than 2.0 mm in the largest dimension. Cases in which at least 1 micrometastasis is detected, but no metastases larger than 2 mm are detected, regardless of number involved, are classified as pN1mi or pN1mi(sn).

^d^In these analyses, node positive was exclusive of the N1mi subgroups. Please refer to Methods section, Data Source subsection for detailed information regarding nodal status classification.

^e^Other indicates histologic subtypes with <1% of patients, and included: phyllodes tumor, Paget disease, inflammatory adenocarcinoma, medullary adenocarcinoma, mucin-producing adenocarcinoma, tubular adenocarcinoma, adenocarcinoma not otherwise specified, epidermoid carcinoma, papillary adenocarcinoma, unspecified carcinoma, other specific carcinoma, unspecified, and other specific types.

Abbreviations: EBC, early breast cancer; HER2, human epidermal growth factor receptor 2; HR, hormone receptor; mi, microinvasive carcinoma; N1, node status; NOS, not otherwise specified; TNBC, triple negative breast cancer.

The mortality analytic cohort included a subset of 228,031 patients with complete data on all risk factors of interest who were included in mortality analyses (**Fig 1**). When compared with the overall population of interest from SEER (ie, the 342,149 patients who met initial inclusion criteria), the mortality analytic cohort tended to be younger, include fewer non-Hispanic White patients, have more patients within the HR+, HER2- subtype, have fewer patients with no nodal involvement, and have more patients with infiltrating duct carcinoma histology (**S2 Table**). Patients who were excluded due to prior cancer diagnoses were also more likely to be diagnosed at a lower stage of disease (perhaps due to greater vigilance given prior cancer). The most common prior cancers among patients excluded for this reason were other primary breast cancer (63.0%), uterine cancer (corpus; 5.4%), and skin cancer (melanoma; 5.4%).

### Breast cancer-specific mortality by HR, HER2 subtype

**Fig 2:**
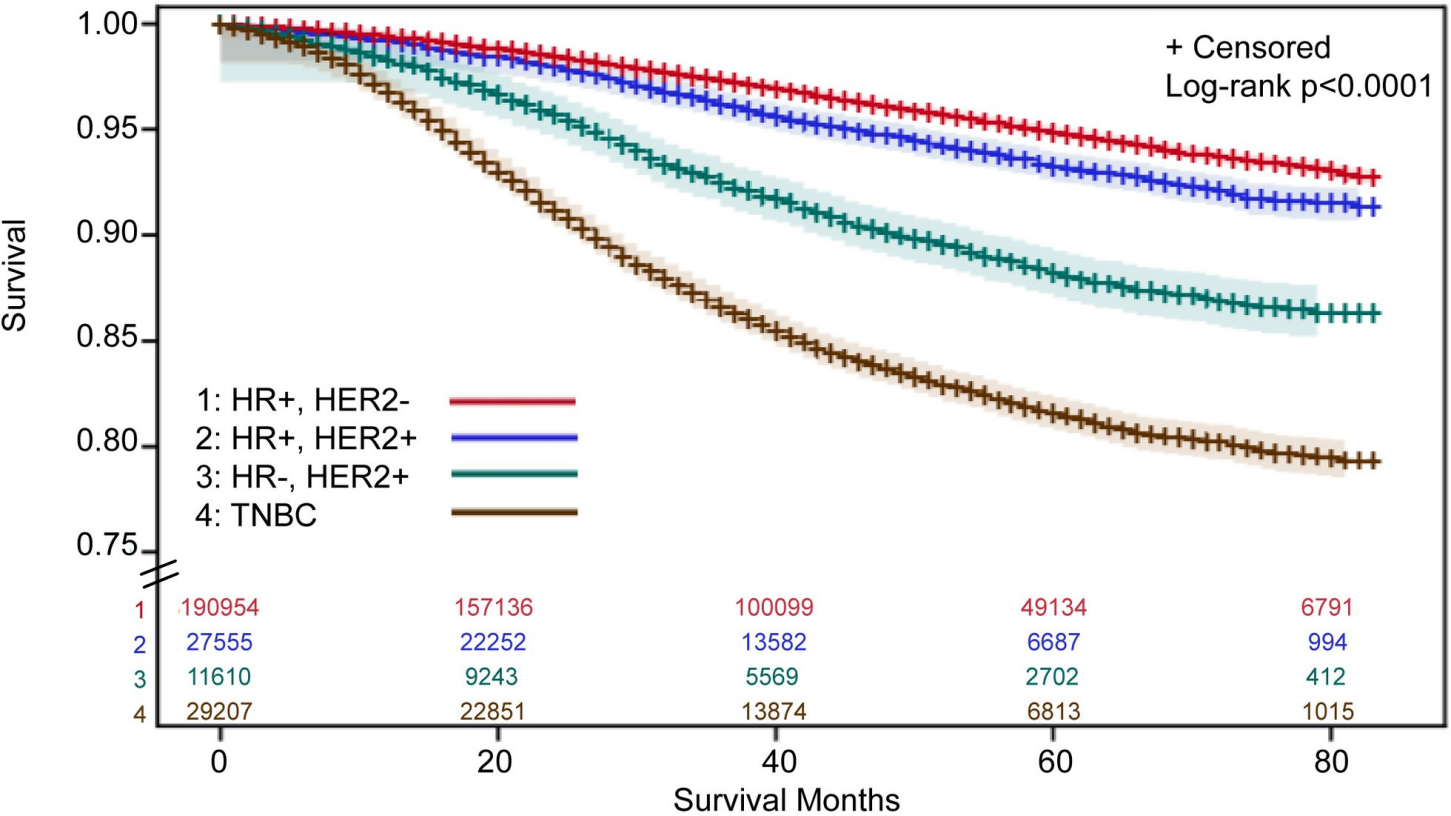
Breast cancer-specific survival estimates^1^ for patients with early breast cancer, by HR, HER2 subtype. ^1^Survival estimates were derived using Kaplan-Meier methods with 95% Hall-Wellner confidence bands. SEER does not provide cancer-specific survival status for patients with a previous tumor, so those patients were excluded. ⸗ Indicates y-axis was truncated to 0.75–1.00. Abbreviations: HER, human epidermal growth factor receptor; HR, hormone receptor; SEER, Surveillance, Epidemiology, and End Results; TNBC, triple negative breast cancer.

Mortality was greatest within the TNBC subtype, followed by HR-, HER2+. Patients with HR+, HER2+ or HR+, HER2- EBC had the lowest risk of mortality (**Fig 2**). When compared with patients with the HR+, HER2- subtype (reference group), after adjusting for other risk factors, including stage, patients with either TNBC (hazard ratio, 2.64; 95% CI, 2.51, 2.78) or HR-, HER2+ (hazard ratio, 1.27; 95% CI, 1.16, 1.38) subtypes had a statistically significant increased risk of mortality (**S3 Table**). Patients with the HR+, HER2+ subtype had a statistically significant decreased risk of mortality, compared with patients with the HR+, HER2- subtype (hazard ratio, 0.83; 95% CI, 0.77, 0.90).

### Relative importance of risk factors

**Fig 3:**
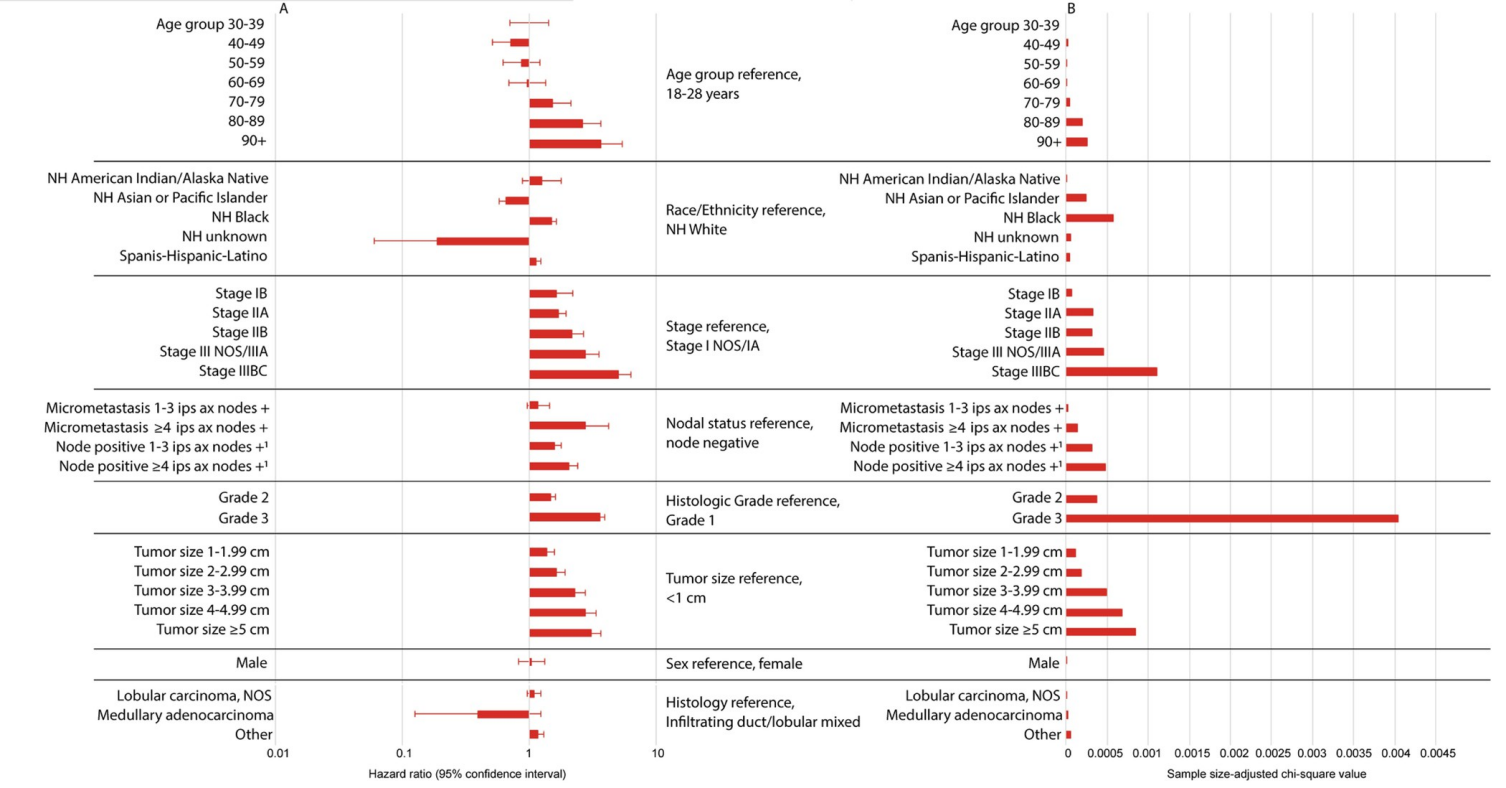
Breast cancer-specific mortality risk factors for patients with HR+, HER2- EBC. Hazard ratios and 95% confidence intervals (A), and sample size-adjusted Chi-square values (B) are presented for each risk factor and were derived from adjusted Cox proportional hazard models. ^1^In these analyses, node positive was exclusive of the N1mi subgroups. Please refer to Materials and Methods section, Data Source subsection for detailed information regarding nodal status classification. Abbreviations: +, positive; AJCC, American Joint Committee on Cancer; Ax, axillary; cm, centimeter; HER, human epidermal growth factor receptor; HR, hormone receptor; Ips, ipsilateral; NH, non-Hispanic; NOS, not otherwise specified.

Histologic Grade 3 (compared with Grade 1) was associated with a significant increased risk of mortality for patients with HR+, HER2- EBC (hazard ratio, 3.61; 95% CI, 3.27, 3.98; p<0.0001) (**Fig 3A**). Relative to all other risk factors examined, within this subtype, histologic Grade 3 was the most influential on mortality, as demonstrated by the largest sample size-adjusted chi-square value (**Fig 3B**).

**Fig 4:**
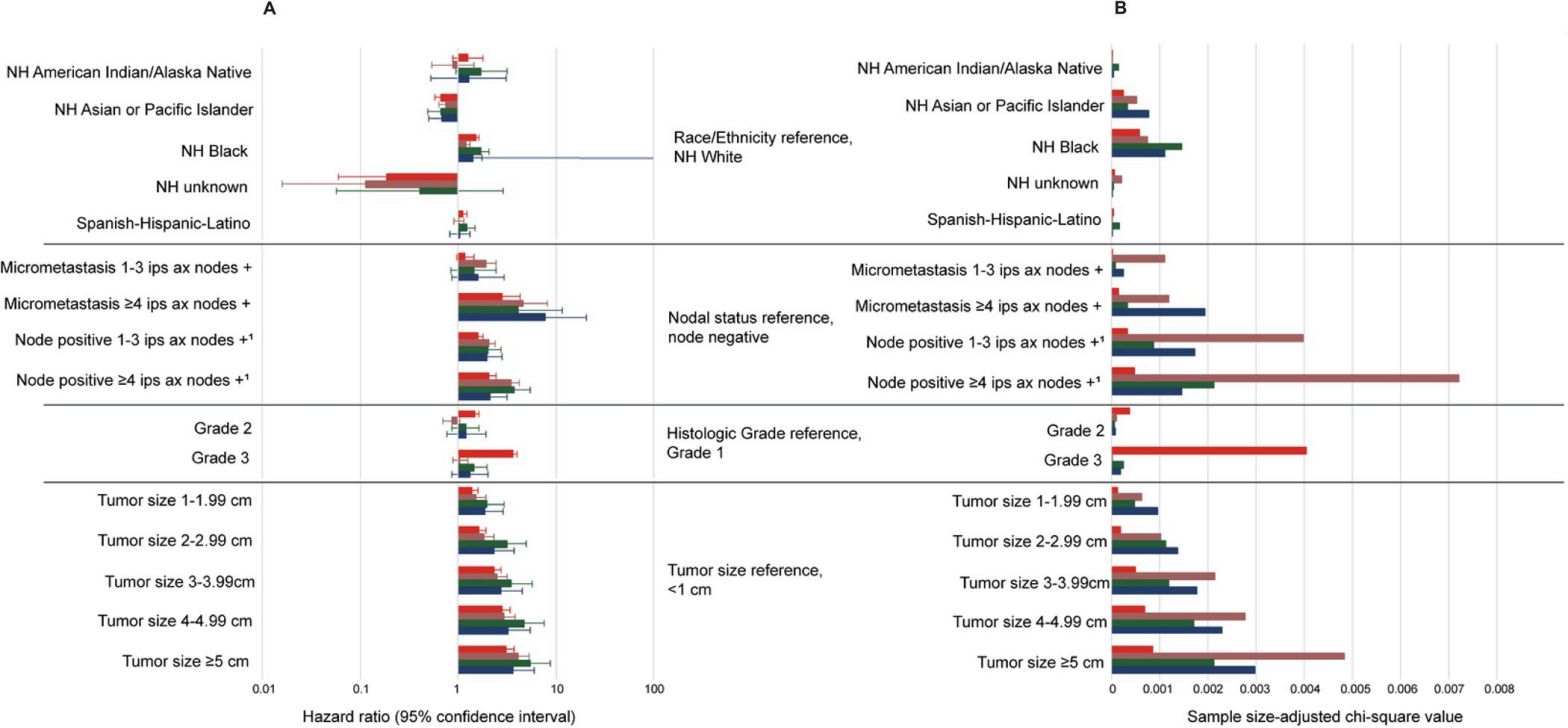
Breast cancer-specific mortality risk factors for patients with EBC, by HR, HER2 subtype. Hazard ratios and 95% confidence intervals (A), and sample size-adjusted Chi-square values (B) are presented for each risk factor and were derived from Cox proportional hazard models. Hazard ratios and sample-size adjusted chi-square values are additionally presented in **S4 Table**. ^1^In these analyses, node positive was exclusive of the N1mi subgroups. Please refer to Materials and Methods section, Data Source subsection for detailed information regarding nodal status classification. Abbreviations: +, positive; AJCC, American Joint Committee on Cancer; Ax, axillary; cm, centimeter; EBC, early breast cancer; HER, human epidermal growth factor receptor; HR, hormone receptor; ips, ipsilateral; NH, non-Hispanic.

For patients within the TNBC subtype, having ≥4 ipsilateral axillary positive nodes (compared with node negative) was associated with a significant increased risk of mortality (hazard ratio, 3.46; 95% CI, 2.87, 4.17; p<0.0001) (**Fig 4A**). Compared with all other risk factors investigated, ≥4 ipsilateral axillary positive nodes had the greatest impact on mortality, for the TNBC subtype, as demonstrated by the largest sample-size adjusted chi-square value (**Fig 4B**).

For patients with HR-, HER2+ EBC, tumor size ≥5 cm (compared with tumor size <1 cm) was associated with a significant increased risk of mortality (hazard ratio, 3.66; 95% CI, 2.23, 6.00; p<0.0001; **Fig 4A**). Having ≥4 positive ipsilateral axillary nodes (compared with node negative) also had a significant association with mortality (hazard ratio, 2.12; 95% CI, 1.40, 3.19; p<0.001; **Fig 4A**). Similar results were found for the HR+, HER2+ subtype in which both tumor size ≥5 cm (compared with tumor size <1 cm; hazard ratio, 5.38; 95% CI, 3.33, 8.70; p<0.0001) and ≥4 positive ipsilateral axillary nodes (compared with node negative; hazard ratio, 3.74; 95% CI, 2.57, 5.44; p<0.0001) were significantly associated with mortality (**Fig 4A**). Relative to all other risk factors, as demonstrated by sample size-adjusted chi-square values, tumor size ≥5 and ≥4 positive ipsilateral axillary nodes were the most influential risk factors on mortality for the HR+, HER2+ and HR-, HER2+ subtypes (**Fig 4B**).

### Breast cancer-specific mortality within the HR+, HER2- subtype

When examining mortality within the HR+, HER2- subtype, Grade 3 was the most influential mortality risk factor in all models (**Table 2**). The only exception was found in Stage III disease, in which advanced age (80–89) was the most influential risk factor on mortality followed by Grade 3 (**Table 2**).

**Table 2:** Hazard ratios (95% confidence intervals) for breast cancer-specific mortality within HR+, HER2- subtype, by Stage.

	Stage I NOS/IA	Stage IB	Stage IIA	Stage IIB	Stage III NOS/IIIA	Stage IIIB-C
	Hazard Ratio (95% CI)					
**Sex (reference, female)**						
Male	2.03 (1.12, 3.69)	0.82 (0.11, 5.99)	0.96 (0.54, 1.70)	1.46 (0.89, 2.39)	1.20 (0.70, 2.04)	0.59 (0.34, 1.02)
**Age group (reference, 18–49)**						
50–59	1.03 (0.77, 1.39)	1.41 (0.73, 2.74)	1.18 (0.96, 1.46)	1.26 (1.05, 1.52)	1.11 (0.92, 1.32)	1.01 (0.85, 1.22)
60–69	1.51 (1.16, 1.97)	1.16 (0.59, 2.31)	1.37 (1.12, 1.69)	1.25 (1.03, 1.52)	1.23 (1.02, 1.48)	1.08 (0.90, 1.29)
70–79	2.62 (2.02, 3.42)	2.58 (1.29, 5.14)	2.28 (1.85, 2.81)	1.73 (1.41, 2.13)	2.10 (1.73, 2.56)	1.43 (1.18, 1.75)
80–89	4.25 (3.16, 5.73)	3.86 (1.75, 8.50)	3.78 (3.00, 4.76)	3.48 (2.78, 4.36)	3.44^a^ (2.75, 4.30)	2.83^a^ (2.31, 3.48)
90+	6.87 (3.16, 14.92)	N/A	3.84 (1.96, 7.53)	6.68 (4.25, 10.49)	6.24 (4.02, 9.69)	2.86 (1.76, 4.66)
**Race/ethnicity (reference, non-Hispanic White)**						
Spanish-Hispanic-Latino	1.08 (0.83, 1.41)	0.87 (0.40, 1.93)	1.17 (0.95, 1.44)	1.12 (0.92, 1.36)	1.22 (1.01, 1.47)	1.06 (0.88, 1.28)
Non-Hispanic Black	1.51 (1.19, 1.91)	1.83 (1.00, 3.34)	1.66 (1.38, 1.99)	1.18 (0.97, 1.42)	1.80 (1.52, 2.13)	1.46 (1.23, 1.73)
Non-Hispanic American Indian/Alaska Native	1.85 (0.88, 3.91)	N/A	1.77 (0.84, 3.73)	0.64 (0.24, 1.72)	1.52 (0.72, 3.20)	0.86 (0.41, 1.81)
Non-Hispanic Asian or Pacific Islander	0.52 (0.35, 0.76)	0.73 (0.26, 2.01)	0.61 (0.45, 0.83)	0.57 (0.43, 0.77)	0.96 (0.75, 1.23)	0.66 (0.50, 0.87)
**Nodal status (reference, node negative)** ^b^						
Micrometastasis 1–3 positive ipsilateral axillary nodes	N/A	N/A	N/A	1.68 (1.15, 2.47)	N/A	1.74 (0.80, 3.82)
Micrometastasis ≥4 positive ipsilateral axillary nodes^c^	N/A	2.02 (0.49, 8.30)	N/A	5.19 (2.54, 10.58)	3.38 (1.59, 7.20)	1.92 (0.45, 8.18)
Node positive 1–3 positive ipsilateral axillary nodes^d^	N/A	N/A	1.34 (0.96, 1.87)	2.15 (1.52, 3.04)	1.83 (1.17, 2.87)	2.39 (1.62, 3.52)
Node positive ≥4 positive ipsilateral axillary nodes^d^	N/A	N/A	1.80 (0.25, 13.12)	6.27 (3.51, 11.21)^e^	2.46 (1.57, 3.85)	2.47 (1.70, 3.58)
**Histologic grade (reference, Grade 1)**						
Grade 2	1.73 (1.43, 2.09)	1.46 (0.83, 2.58)	1.32 (1.08, 1.61)	1.69 (1.31, 2.18)	1.21 (0.97, 1.51)	1.24 (0.98, 1.56)
Grade 3	4.72^a^ (3.83, 5.83)	4.10^a^ (2.23, 7.53)	3.56^a^ (2.91, 4.36)	4.25^a^ (3.30, 5.47)	2.77 (2.21, 3.47)	2.78 (2.20, 3.51)
**Tumor size (reference, <1 cm)** ^**f**^						
≥1 cm to <2 cm	1.56 (1.30, 1.87)	1.08 (0.60, 1.94)	0.80 (0.58, 1.12)	N/A	0.84 (0.47, 1.51)	1.22 (0.67, 2.23)
≥2 cm to <3 cm	2.63 (1.99, 3.48)	1.12 (0.47, 2.69)	0.93 (0.60, 1.43)	N/A	1.15 (0.65, 2.03)	0.87 (0.48, 1.58)
≥3 cm to <4 cm	N/A	N/A	1.41 (0.88, 2.26)	1.48 (1.26, 1.73)	1.45 (0.82, 2.56)	1.33 (0.74, 2.38)
≥4 cm to <5 cm	N/A	N/A	1.65 (1.01, 2.69)	1.98 (1.65, 2.37)	1.82 (1.02, 3.23)	1.42 (0.79, 2.56)
≥5 cm	N/A	N/A	1.40 (0.71, 2.76)	2.64 (1.95, 3.58)	2.05 (1.17, 3.59)	1.80 (1.01, 3.19)
**Histology (reference, infiltrating duct/lobular mixed)**						
Lobular carcinoma, NOS	0.99 (0.67, 1.46)	1.28 (0.43, 3.83)	1.40 (1.03, 1.91)	0.80 (0.59, 1.08)	0.88 (0.69, 1.13)	1,27 (1.00, 1.60)
Other^g^	1.26 (0.96, 1.66)	1.27 (0.58, 2.78)	1.49 (1.17, 1.91)	1.22 (0.98, 1.53)	1.00 (0.82, 1.23)	1.05 (0.85, 1.28)

^a^Indicates highest chi-square value within each Stage.

^b^Reference group for Stage IB and Stage III NOS/IIIA is “Micrometastasis 1–3 Ips Ax nodes+”.

^c^Per the *American Joint Committee on Cancer Staging Manual*, micrometastases were defined as tumor deposits larger than 0.2 mm but not larger than 2.0 mm in the largest dimension. Cases in which at least 1 micrometastasis is detected, but no metastases larger than 2 mm are detected, regardless of number involved are classified as pN1mi or pN1mi(sn).

^d^In these analyses, node positive was exclusive of the N1mi subgroups. Please refer to Materials and Methods section, Data Source subsection for detailed information regarding nodal status classification.

^e^It is unclear if patients with Stage IIB tumors and with ≥4 positive ipsilateral axillary nodes were staged clinically, staged by proposed prognostic staging criteria (AJCC 8^th^ Edition), or were staged incorrectly.

^f^Reference group for Stage IIB is “Size ≥2 cm to <3 cm”.

^g^Other combines other adenocarcinomas, mucinous adenocarcinoma, and histologic subtypes with <1% of patients which included: phyllodes tumor, Paget disease, inflammatory adenocarcinoma, medullary adenocarcinoma, mucin-producing adenocarcinoma, tubular adenocarcinoma, adenocarcinoma not otherwise specified, epidermoid carcinoma, papillary adenocarcinoma, unspecified carcinoma, other specific carcinoma, unspecified, and other specific types.

Abbreviations: CI, confidence interval; HER2, human epidermal growth factor receptor 2; HR, hormone receptor; mi, microinvasive carcinoma; N1, node status; N/A, not applicable; NOS, not otherwise specified.

Among patients with HR+, HER2- EBC, nodal positivity, specifically having ≥4 positive ipsilateral axillary nodes, was also associated with increased risk of mortality (**Table 2**). Of note, 120 (0.1%) patients had disease classified as Stage IIB but had ≥4 positive ipsilateral axillary nodes (N2). Among these patients, having ≥4 positive ipsilateral axillary nodes was also associated with increased risk of mortality (hazard ratio, 6.27; 95% CI, 3.51, 11.21). It is unclear if patients with Stage IIB tumors and ≥4 positive ipsilateral axillary nodes were staged clinically, staged by proposed prognostic staging criteria (*AJCC Cancer Staging Manual*, 8^th^ edition), or were staged incorrectly. Also, of note is the increased risk of mortality associated with micrometastasis in ≥4 positive ipsilateral axillary nodes, particularly for patients who had disease classified as Stage IB (n = 57, hazard ratio, 2.02; 95% CI, 0.49, 8.30), Stage IIB (n = 88, hazard ratio, 5.19; 95% CI, 2.54, 10.58), and Stage III NOS/IIIA (n = 47; hazard ratio, 3.38; 95% CI, 1.59, 7.20). This contrasts with the small increased risk of mortality for patients who had Stage IIB disease and 1–3 positive ipsilateral axillary micrometastasis (n = 69; hazard ratio, 1.68; 95% CI, 1.15, 2.47) (**Table 2**).

Within the HR+, HER2- subtype, other risk factors with a statistically significant association with risk of mortality, across all disease Stages, included age 70–79, 80–89 and 90+ years (each compared with a reference group of 18–49 years). Being non-Hispanic Black (compared with a reference group of non-Hispanic White) was also associated with a statistically significant increased risk of mortality for patients classified with Stage I NOS/IA, Stage IIA, Stage III NOS/IIIA, and Stage IIIB-C disease (**Table 2**).

### Comparison of patients who did and did not meet monarchE clinicopathologic high-risk criteria

Among patients with HR+, HER2- EBC, 28,619 (12.0%) did and 209,603 (88.0%) did not meet clinicopathological high-risk criteria set forth in the monarchE clinical trial (**Table 3**). The percentage of patients who were Spanish-Hispanic-Latino was larger in the group who met monarchE clinicopathologic high-risk criteria (13.5%) versus those who did not (Stage II-III/node positive, 12.5% and Stage I and/or node negative, 9.3%). There was also a larger percentage of patients who were non-Hispanic Black in the group who met monarchE clinicopathological high-risk criteria (12.3%) compared with those who did not (Stage II-III/node positive, 10.4% and Stage I and/or node negative, 8.1%) (**Table 3**). Additionally, there was a larger percentage of patients in the younger age groups (18–29, 30–39, 40–49, and 50–59) in the group who met monarchE clinicopathological high-risk criteria compared with those who did not, while the percentage of patients ≥60 years was larger in the group who did not meet monarchE clinicopathological high-risk criteria (**Table 3**).

**Table 3:** Characteristics of patients within the HR+, HER2- subtype, stratified by monarchE clinicopathological high-risk inclusion criteria^a^.

N (%), unless otherwise specified	Did Not Meet monarchE Criteria^a^		Did Meet monarchE Criteria^a^
	n = 209 603 (88.0%)		n = 28 619 (12.0%)
	Stage II-III/Node Positive n = 30 041 (14.3%)	Stage I and/or Node Negative n = 179 562 (85.7%)	
**Sex**			
Male	338 (1.1)	1 289 (0.7)	414 (1.5)
Female	29 703 (98.9)	178 273 (99.3)	28 205 (98.6)
**Age, mean (SD)**	60.3 (13.2)	63.7 (12.9)	58.5 (13.7)
**Age group**			
18–29	137 (0.5)	410 (0.2)	227 (0.8)
30–39	1 322 (4.4)	3 939 (2.2)	2 002 (7.0)
40–49	5 432 (18.1)	22 983 (12.8)	5 848 (20.4)
50–59	7 639 (25.4)	39 119 (21.8)	7 333 (25.6)
60–69	8 123 (27.0)	52 654 (29.3)	6 949 (24.3)
70–79	4 807 (16.0)	38 847 (21.6)	4 072 (14.2)
80–89	2 260 (7.5)	18 806 (10.5)	1 919 (6.7)
90+	321 (1.1)	2 804 (1.6)	269 (0.9)
**Race/ethnicity**			
Spanish-Hispanic-Latino	3 768 (12.5)	16 653 (9.3)	3 852 (13.5)
Non-Hispanic White	20 417 (68.0)	131 847 (73.4)	18 602 (65.0)
Non-Hispanic Black	3 108 (10.4)	14 619 (8.1)	3 520 (12.3)
Non-Hispanic American Indian/Alaska Native	174 (0.6)	904 (0.5)	176 (0.6)
Non-Hispanic Asian or Pacific Islander	2 438 (8.1)	14 730 (8.2)	2 373 (8.3)
Non-Hispanic unknown	136 (0.5)	809 (0.5)	96 (0.3)
**Stage**			
Stage I NOS/IA	N/A	132 575 (73.8)	N/A
Stage IB	N/A	6 273 (3.5)	N/A
Stage IIA	12 975 (43.2)	35 411 (19.7)	2 564 (9.0)
Stage IIB	14 758 (49.1)	3 862 (2.2)	6 292 (22.0)
Stage III NOS/IIIA	811 (2.7)	159 (0.1)	13 218 (46.2)
Stage IIIB-C	1 497 (5.0)	1 282 (0.7)	6 545 (22.9)
**Nodal status**			
Node negative		173 289 (96.5)	
Micrometastasis 1–3 positive ipsilateral axillary nodes	2 767 (10.0)	6 140 (3.4)	1 617 (5.7)
Micrometastasis ≥4 positive ipsilateral axillary nodes^b^	N/A	71 (0.04)	188 (0.7)
Node positive 1–3 positive ipsilateral axillary nodes^c^	24 845 (90.0)	N/A	11 919 (41.7)
Node positive ≥4 positive ipsilateral axillary nodes^c^	N/A	N/A	14 895 (52.1)
**Histologic grade**			
Grade 1	7 361 (24.5)	63 789 (35.5)	2 676 (9.4)
Grade 2	18 778 (62.5)	79 566 (44.3)	9 820 (34.3)
Grade 3	670 (2.2)	23 438 (13.1)	14 459 (50.5)
Grade unknown	3 232 (10.8)	12 769 (7.1)	1 664 (5.8)
**Tumor size**			
<1 cm	2 231 (7.4)	54 990 (30.6)	713 (2.5)
≥1 cm to <2 cm	9 647 (32.1)	77 245 (43.0)	4 263 (14.9)
≥2 cm to <3 cm	9 765 (32.5)	28 451 (15.8)	6 746 (23.6)
≥3 cm to <4 cm	4 741 (15.8)	9 515 (5.3)	4 521 (15.8)
≥4 cm to <5 cm	2 569 (8.6)	4 105 (2.3)	2 769 (9.7)
≥5 cm	779 (2.6)	4 982 (2.8)	9 288 (32.5)
Tumor size unknown	309 (1.0)	274 (0.2)	319 (1.1)
**Laterality**			
Left	14 762 (49.1)	88 914 (49.5)	14 222 (49.7)
Right	15 264 (50.8)	90 622 (50.5)	14 383 (50.3)
Other	15 (0.1)	26 (0.01)	14 (0.1)
**Histology**			
Other adenocarcinomas	302 (1.0)	1 668 (0.9)	368 (1.3)
Mucinous adenocarcinoma	261 (0.9)	5 605 (3.1)	153 (0.5)
Infiltrating duct carcinoma	21 481 (71.5)	128 148 (71.4)	19 726 (68.9)
Lobular carcinoma, NOS	3 730 (12.4)	20 001 (11.1)	4 541 (15.9)
Infiltrating duct mixed/infiltrating lobular mixed	3 685 (12.3)	19 335 (10.8)	3 231 (11.3)
Other^d^	582 (1.9)	4 805 (2.7)	600 (2.1)

^a^Without Ki-67 ≥20%, as these data were not available in the SEER database.

^b^Per the American Joint Committee on Cancer Staging Manual, micrometastases were defined as tumor deposits larger than 0.2 mm but not larger than 2.0 mm in the largest dimension. Cases in which at least 1 micrometastasis is detected, but no metastases larger than 2 mm are detected, regardless of number involved are classified as pN1mi or pN1mi(sn).

^c^In these analyses, node positive was exclusive of the N1mi subgroups. Please refer to Materials and Methods section, Data Source subsection for detailed information regarding nodal status classification.

^d^Other indicates histologic subtypes with <1% of patients overall, and included: phyllodes tumor, Paget disease, inflammatory adenocarcinoma, medullary adenocarcinoma, mucin-producing adenocarcinoma, tubular adenocarcinoma, adenocarcinoma not otherwise specified, epidermoid carcinoma, papillary adenocarcinoma, unspecified carcinoma, other specific carcinoma, unspecified, and other specific types.

Abbreviations: HER2, human epidermal growth factor receptor 2; HR, hormone receptor; mi, microinvasive carcinoma; N1, node status; N/A, not applicable; NOS, not otherwise specified; SD, standard deviation.

**Fig 5:**
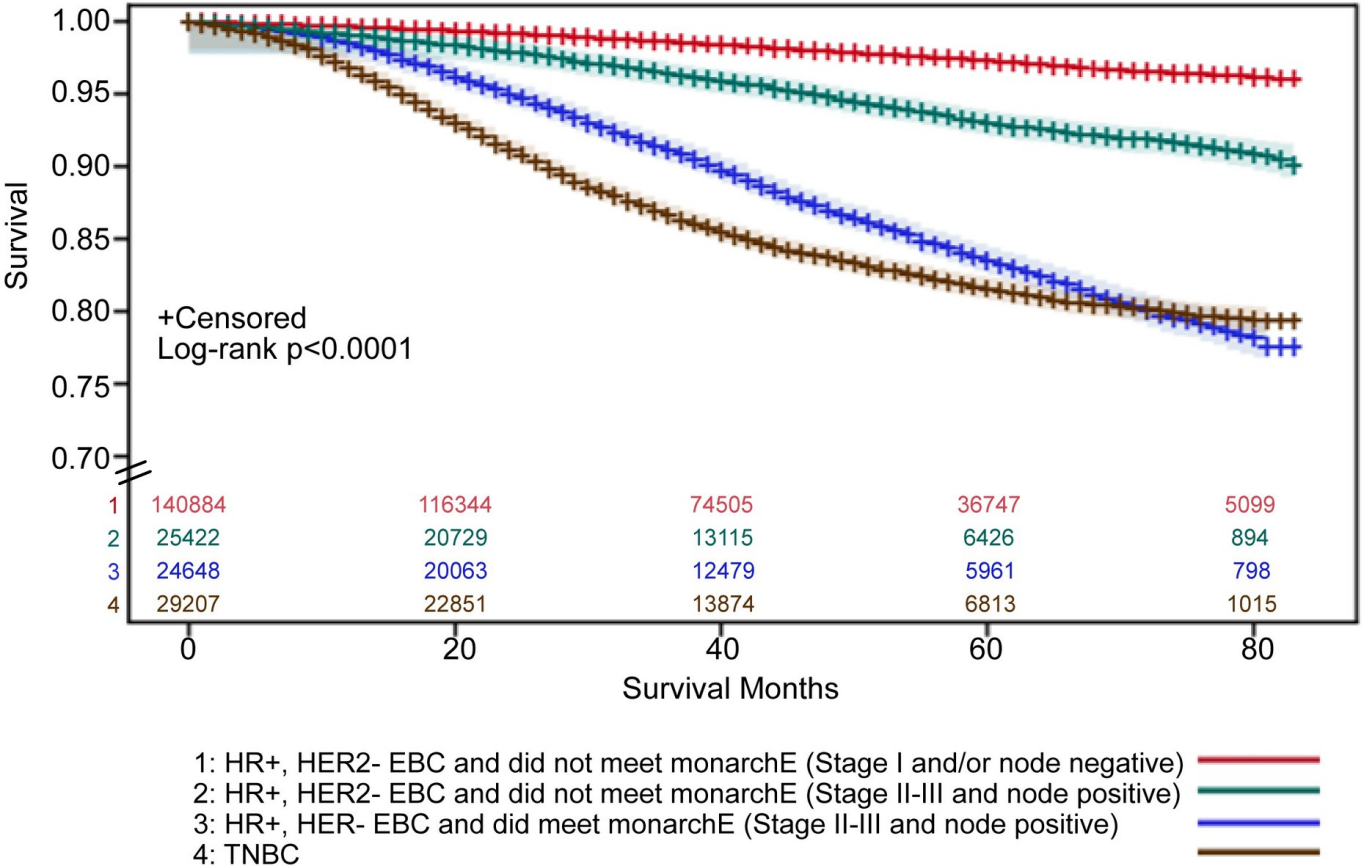
Survival estimates^1^ for patients with HR+, HER2- EBC who did/did not meet monarchE clinicopathological high-risk criteria. ^**2** 1^Kaplan-Meier estimates of survival were calculated with 95% Hall-Wellner confidence bands. ^2^Without Ki-67 index ≥20%, as these data were not available in the SEER database. ⸗ Indicates y-axis was truncated to 0.70–1.00. Abbreviations: EBC, early breast cancer; HER, human epidermal growth factor receptor; HR, hormone receptor; SEER, Surveillance, Epidemiology, and End Results; TNBC, triple negative breast cancer.

Using Kaplan-Meier survival estimates, mortality (adjusted for age, sex, race/ethnicity) was greatest among patients with HR+, HER2- EBC who met the monarchE clinicopathological high-risk criteria and all patients with early TNBC (**Fig 5**). The 60-month mortality rate (adjusted for age, sex, race/ethnicity) for patients who met monarchE clinicopathological inclusion criteria was 16.5%, compared with 7.0% who did not meet the monarchE clinicopathological high-risk criteria among similar Stage II-IIIC and node positive patients, and 2.8% within the Stage I and/or node negative subgroup (**Fig 5**). Comparatively, the 60-month mortality rate for all patients with early TNBC was 18.5% (**Fig 5**). In Cox proportional hazard models, compared with patients who did not meet monarchE clinicopathological high-risk criteria, patients who did meet monarchE clinicopathological high-risk criteria had a higher risk of mortality (hazard ratio, 2.58; 95% CI: 2.41, 2.76).

### Incidence of monarchE clinicopathological high-risk criteria in the SEER cohort

Of the 342,149 patients with EBC (Stage I–IIIC) in the SEER cohort, 238,222 (69.6%) patients had HR+, HER2- EBC. Of these, 58,660 patients (24.6%) had node positive EBC, and of these, 28,619 (48.8%) met monarchE clinicopathological high-risk criteria (**S1 Fig**). Among patients with HR+, HER2- node positive EBC, 15,083 (25.7%) met the monarchE criterion of ≥4 positive ALNs and 13,536 (23.1%) met the criteria of 1–3 positive ALNs in combination with either a primary invasive tumor size ≥5 cm or histological Grade 3 tumor (**S1 Fig**). Among patients with HR+, HER2- EBC, 28,619 (12.0%) met monarchE clinicopathological high-risk criteria.

## Discussion

This study describes the US population of patients with EBC included in the SEER 2010–2015 registry data and the risk of breast cancer-specific mortality. Among patients with HR+, HER2- EBC from SEER, 12.0% met the clinicopathological criteria for high risk of early recurrence used in the monarchE clinical trial. Consistent with expectations [2], these data confirmed that patients with HR-, HER2+ or early TNBC have a disproportionately greater risk of breast cancer-specific mortality compared with HR+ EBC. Strikingly, results further demonstrated that patients with HR+, HER2- EBC who met monarchE clinicopathological high-risk criteria were at a statistically significant increased risk of mortality in this 5-year period compared with patients who did not meet monarchE clinicopathological high-risk criteria, and were at nearly the same risk of mortality as patients with early TNBC. Patients with HR+, HER2- EBC who met the monarchE clinicopathological high-risk criteria had a 60-month mortality rate (16.5%), which was very similar to the rate in patients with early TNBC (18.5%). It is important to highlight that patients with HR+, HER2- EBC who met monarchE clinicopathological high-risk criteria made up 8.4% of the total EBC population while TNBC comprised 10.5% of the total EBC population. While there is awareness of high unmet need for therapeutic advancements for patients with TNBC, similar discussions are not common for patients with HR+, HER2- EBC. Cumulatively, the data presented here suggest there is also a high unmet need for therapeutic advancements among patients with HR+, HER2- EBC at a high risk of recurrence. Furthermore, these data independently demonstrate that the clinicopathological high-risk inclusion criteria used in monarchE [19, 20], identify patients with HR+, HER2- EBC that have a poor prognosis and are candidates for additional therapies to improve outcomes.

This study further investigated the relative importance of risk factors for mortality by breast cancer subtype, with the goal of identifying groups of patients who may be at the highest risk for recurrence and who may benefit most from therapy escalation. Results for the TNBC, HR+, HER2- and HR+, HER2+ subtypes were aligned with previous studies highlighting factors such as nodal status and tumor size as key risk factors for recurrence [4, 29]. We noted that patients with TNBC and HR-, HER2+ EBC were often diagnosed at later Stages. Sample sizes were not large enough to permit analyses by Stage for these subtypes. However, mortality analyses did take Stage into account, suggesting that the increased mortality associated with these subtypes is due to more than advanced Stage at diagnosis.

Nodal status was not the most influential risk factor for the HR+, HER2- subtype, where histologic Grade 3 had a greater influence on mortality. These results suggest biology, as reflected by high histologic Grade, may be an even greater prognostic factor than nodal status and tumor size in making treatment decisions for patients with high-risk HR+, HER2- EBC. Consistent with our results, the *AJCC Cancer Staging Manual*, 8^th^ edition [30] recognized the importance of histologic Grade as a biological factor in prognosis and was included in the proposed prognostic staging. Cumulatively, results support the continued use of Grade and other biological factors for informing treatment decisions within the HR+, HER2- subtype, such as multi-gene assays and Ki-67 level, which have been associated with disease-free survival, overall survival, and risk of recurrence [8–12, 31]. One limitation to these analyses is that SEER did not contain variables to describe the tumoral microenvironment, such as tumor stroma ratio/tumor stromal type, myxoid change, or fibrotic focus, which have all recently been shown to be associated with mortality in breast cancer, particularly in TNBC [32–34]. Further investigations that assess the variables reported here in combination with the tumoral microenvironment are needed, to determine if relative importance of risk factors remains the same.

In a pre-planned analysis of the adjuvant monarchE trial, patients with tumors that had high clinical risk factors examined here and high Ki-67 levels (≥20%) had a poor prognosis in the ET-only arm despite conventional treatment to include chemotherapy and ET (2-year invasive disease-free survival rate 86.1% [83.1, 88. 7] vs. 92.0% [89.7, 93.9] in patients with high clinical risk factors and tumors with Ki-67 levels [<20%]) [35]. While patients with high or low Ki-67 (based on a 20% cut off) benefitted from the addition of abemaciclib to ET in monarchE, patients with high risk clinical and pathological factors and high Ki-67 had an even higher risk of recurrence suggesting that Ki-67 is a useful additional prognostic factor [35]. More recent results from monarchE further confirmed that Ki-67 was prognostic, and that the abemaciclib benefit was observed independent of Ki-67 level and beyond the 2-year treatment period [20]. At 27 months median follow-up, with 90% of patients off study drug, abemaciclib treatment benefit was maintained and reflected in the reduction in the risk of an IDFS or DRFS event by 30% (hazard ratio = 0.70, 95% CI 0.59–0.82; nominal p<0.0001) and 31% (hazard ratio = 0.69, 95% CI 0.57–0.83; nominal p<0.0001), respectively [20]. The absolute improvements in 3-year IDFS and DRFS rates were 5.4% and 4.2%, respectively [20]. A limitation of the current study was that it was not possible to assess the potential prognostic value of Ki-67, or other potential markers reflecting underlying tumor biology, such as multi-gene assays, as these were not included in the SEER registry.

While nodal status did not exert the greatest influence on mortality risk among patients with HR+, HER2- EBC, some interesting results did emerge regarding nodal involvement within this subtype. The *AJCC Cancer Staging Manual*, 8th edition [30] defines micrometastases as tumor deposits larger than 0.2 mm but not larger than 2.0 mm in the largest dimension. For cases in which at least one micrometastasis is detected, but no metastases larger than 2 mm are detected, regardless of number of nodes involved, are classified as microinvasive carcinoma (pN1mi). We found a distinct difference in risk of mortality between patients with Stage IIB HR+, HER2- EBC who had micrometastases with 1–3 positive ipsilateral axillary nodes and those patients who had micrometastases with ≥4 positive ipsilateral axillary nodes. The latter had nearly the same level of mortality risk as those patients who had macrometastases with ≥4 ipsilateral axillary nodes. These results may be informative for assessing risk associated with lymph node micrometastases versus macrometastases and warrant further confirmation.

Race and ethnicity emerged from these analyses as an important risk factor to consider. Not only are non-Hispanic Black and Spanish-Hispanic-Latino patients more likely to be diagnosed with the HR- subtypes that are associated with increased risk of mortality, but they were also more likely to meet the high-risk criteria within the HR+, HER2- subtype. While only a small portion of patients with HR+, HER2- EBC (12.0%) met the monarchE-defined high-risk criteria, patients who were of racial/ethnic minority groups were more likely to meet these high-risk criteria. These results highlight a potential racial disparity in the unmet need of patients with HR+, HER2- EBC.

Some limitations should be considered when interpreting the results from this study. Data used in these analyses were from the 2010–2015 SEER registry and thus may be slightly outdated (although this represents the most recent verified data available in SEER). While cancer registries participating in the SEER program cover approximately 34% of the US population, these data may not be representative of the entire population. Due to missing data, different analytic samples were used for different analyses, so results cannot be directly compared. Analyses did not adjust for comorbidities, nor treatment (including chemotherapy), as these data were not available in SEER.

## Conclusions

In spite of treatment advances in the last decade, there is still a large unmet need among patients with EBC who experience distant relapse and will invariably die from this disease. Patients with early TNBC have long been recognized as a subgroup with the largest risk for recurrence and death. These data demonstrate there is also a high unmet need for therapeutic advancements among select patients with HR+, HER2- EBC. Patients with HR+ EBC and a select group of high-risk factors, including histologic Grade 3, tumor size ≥5 cm, and ≥4 positive ipsilateral axillary nodes are at nearly the same level of risk for breast cancer-specific mortality. These data highlight US patients with EBC who may benefit most from therapy escalation. Future studies should confirm these associations, examine these associations with an outcome of recurrence, and elaborate on risk factors, including potential biological markers. Finally, future studies are needed to confirm that patients with micrometastases and ≥4 lymph nodes involved do as poorly as those with macroscopic lymph node disease.

## Supporting information

S1 Text Supplementary methods. Details regarding controlling for multiplicity.
(DOCX)

S1 Fig Percentage of patients at high risk of breast cancer-specific mortality in the invasive breast cancer population, by subtype and nodal status, derived from SEER 2010–2015. ^1^Excludes 6273 HR+, HER2- Stage I-IIIC patients with N1mi+ Stage IB (not considered high-risk). High risk was based on the monarchE criteria, without Ki-67 index ≥20% because that data was not available in the SEER database. Percentages are shown above the box to which they are applicable and were calculated as the number of patients who met the criteria in that box, out of the total presented in the prior level. Abbreviations: cm, centimeter; EBC, early breast cancer; HER, human epidermal growth factor receptor; HR, hormone receptor; N+, node positive.
(TIF)

S1 Table Detailed list of variables and risk factors of interest extracted from SEER. Abbreviations: AJCC-7, *American Joint Committee on Cancer Staging Manual*, 7^th^ edition; CS, collaborative stage; HER, human epidermal growth factor receptor; HR, hormone receptor; ICD-O-3, International Classification of Diseases for Oncology, 3^rd^ edition; NAACCR, The North American Association of Central Cancer Registries; NHIA, The NAACCR Hispanic/Latino Identification Algorithm; SEER, Surveillance, Epidemiology, and End Results Program.
(DOCX)

S2 Table Demographic and clinical characteristics of the overall population of interest from SEER who met initial inclusion criteria, the mortality analytic cohort, and those excluded from the mortality analytic cohort. ^a^Chi-square p-value of mortality analytic cohort versus excluded cohort. ^b^Per the *American Joint Committee on Cancer Staging Manual*, micrometastases were defined as tumor deposits larger than 0.2 mm but not larger than 2.0 mm in the largest dimension. Cases in which at least 1 micrometastasis is detected, but no metastases larger than 2 mm are detected, regardless of number involved are classified as pN1mi or pN1mi(sn). ^c^In these analyses, node positive was exclusive of the N1mi subgroups. Please refer to Materials and Methods section, Data Source subsection for detailed information regarding nodal status classification. ^d^Other combines histologic subtypes with <1% of patients which included: phyllodes tumor, Paget disease, inflammatory adenocarcinoma, medullary adenocarcinoma, mucin-producing adenocarcinoma, tubular adenocarcinoma, adenocarcinoma not otherwise specified, epidermoid carcinoma, papillary adenocarcinoma, unspecified carcinoma, other specific carcinoma, unspecified, and other specific types. Abbreviations: HER2, human epidermal growth factor receptor 2; HR, hormone receptor; mi, microinvasive carcinoma; N1, node status; NOS, not otherwise specified; TNBC, triple negative breast cancer.
(DOCX)

S3 Table Multivariable Cox proportional hazards regression results for breast cancer-specific mortality. ^a^Not statistically significant; indicates p≥0.05 in either the model building and/or model validation halves. ^b^Significant at p<0.05 level in the model building and model validation halves, and p<0.0001 in the overall model. ^c^Per the *American Joint Committee on Cancer Staging Manual*, micrometastases were defined as tumor deposits larger than 0.2 mm but not larger than 2.0 mm in the largest dimension. Cases in which at least 1 micrometastasis is detected, but no metastases larger than 2 mm are detected, regardless of number involved are classified as pN1mi or pN1mi(sn). ^d^In these analyses, node positive was exclusive of the N1mi subgroups. Please refer to Materials and Methods section, Data Source subsection for detailed information regarding nodal status classification. ^e^Other combines other adenocarcinomas, mucinous adenocarcinoma, and histologic subtypes with <1% of patients which included: phyllodes tumor, Paget disease, inflammatory adenocarcinoma, medullary adenocarcinoma, mucin-producing adenocarcinoma, tubular adenocarcinoma, adenocarcinoma not otherwise specified, epidermoid carcinoma, papillary adenocarcinoma, unspecified carcinoma, other specific carcinoma, unspecified, and other specific types. Abbreviations: CI, confidence interval; HER2, human epidermal growth factor receptor 2; HR, hormone receptor; mi, microinvasive carcinoma; N1, node status; NOS, not otherwise specified; TNBC, triple negative breast cancer.
(DOCX)

S4 Table Hazard ratio and sample-size adjusted chi-square values for breast cancer-specific mortality risk factors by HR, HER2 subtype. ^a^Per the *American Joint Committee on Cancer Staging Manual*, micrometastases were defined as tumor deposits larger than 0.2 mm but not larger than 2.0 mm in the largest dimension. Cases in which at least 1 micrometastasis is detected, but no metastases larger than 2.0 mm are detected, regardless of number involved are classified as pN1mi or pN1mi(sn). ^b^In these analyses, node positive was exclusive of the N1mi subgroups. Please refer to Materials and Methods section, Data Source subsection for detailed information regarding nodal status classification. ^c^Other combines other adenocarcinomas, mucinous adenocarcinoma, and histologic subtypes with <1% of patients which included: phyllodes tumor, Paget disease, inflammatory adenocarcinoma, medullary adenocarcinoma, mucin-producing adenocarcinoma, tubular adenocarcinoma, adenocarcinoma not otherwise specified, epidermoid carcinoma, papillary adenocarcinoma, unspecified carcinoma, other specific carcinoma, unspecified, and other specific types. Abbreviations: HER2, human epidermal growth factor receptor 2; HR, hormone receptor; mi, microinvasive carcinoma; N1, node status; NE, not estimable; NOS, not otherwise specified; TNBC, triple negative breast cancer.
(DOCX)
