# Supplementary material for: Breast cancer-specific mortality in early breast cancer as defined by high-risk clinical and pathologic characteristics
Source: PLoS One. 2022 Feb 25;17(2):e0264637. doi: 10.1371/journal.pone.0264637 (PMC8880870; doi:10.1371/journal.pone.0264637)
Supplement: S1 Text — Details regarding controlling for multiplicity. (DOCX) [file pone.0264637.s001.docx]

**S1 Text. Supplementary methods.** *Details regarding controlling for multiplicity.*
Type I error was controlled 2 ways. The primary objectives were based on 6 hypothesis tests among the validation half of the data (three 2-way interactions of breast subtype with Grade, nodal status, and tumor size; 3 tests whether Grade 3 was more influential in HR+, HER2- versus other 3 subtypes), p<0.008 indicated statistical significance (p<0.05/6) for the interactions. The data was randomly divided into 2 equal subsets, and the model building subset was used to examine main effects and interactions. Therefore, the tests of the three 2-way interactions are based on the model validation subset. In addition, once the Cox proportional-hazards model for cancer-specific mortality was built within the initial subset, it was also tested in the independent model validation subset. Only risk factors of interest that were significant (p<0.05 2-tailed) in both the model building and data validation halves were considered statistically significant, providing an overall probability of 0.045 that any truly non-statistically significant variables, or type I errors, were observed to be statistically significant in the final model.
