# Supplementary figures and images for: Breast cancer-specific mortality in early breast cancer as defined by high-risk clinical and pathologic characteristics
Source: PLoS One. 2022 Feb 25;17(2):e0264637. doi: 10.1371/journal.pone.0264637 (PMC8880870; doi:10.1371/journal.pone.0264637)

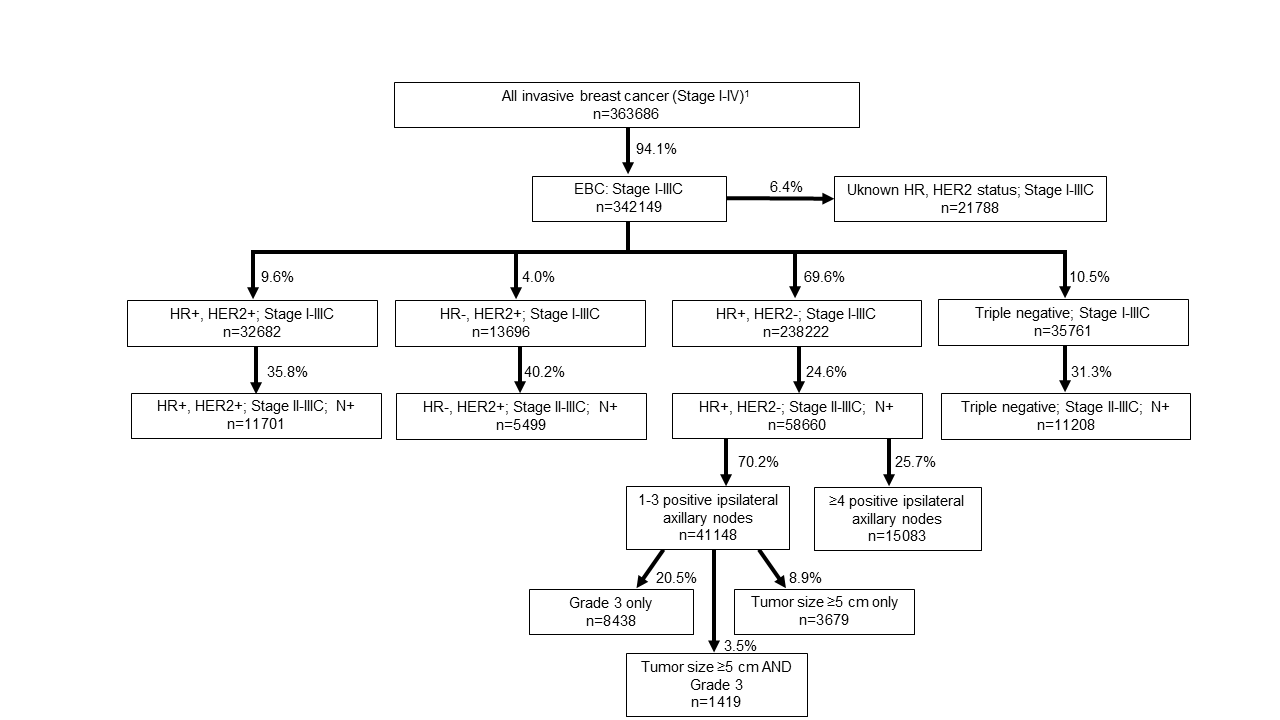

Supplement: S1 Fig — 1Excludes 6273 HR+, HER2- Stage I-IIIC patients with N1mi+ Stage IB (not considered high-risk). High risk was based on the monarchE criteria, without Ki-67 index ≥20% because that data was not available in the SEER database. Percentages are shown above the box to which they are applicable and were calculated as the number of patients who met the criteria in that box, out of the total presented in the prior level. Abbreviations: cm, centimeter; EBC, early breast cancer; HER, human epidermal growth factor receptor; HR, hormone receptor; N+, node positive. (TIF) [file pone.0264637.s002.tif]
