# Supplementary material for: Breast cancer-specific mortality in early breast cancer as defined by high-risk clinical and pathologic characteristics
Source: PLoS One. 2022 Feb 25;17(2):e0264637. doi: 10.1371/journal.pone.0264637 (PMC8880870; doi:10.1371/journal.pone.0264637)
Supplement: S1 Table — Abbreviations: AJCC-7, American Joint Committee on Cancer Staging Manual, 7th edition; CS, collaborative stage; HER, human epidermal growth factor receptor; HR, hormone receptor; ICD-O-3, International Classification of Diseases for Oncology, 3rd edition; NAACCR, The North American Association of Central Cancer Registries; NHIA, The NAACCR Hispanic/Latino Identification Algorithm; SEER, Surveillance, Epidemiology, and End Results Program. (DOCX) [file pone.0264637.s003.docx]

**S1 Table. Detailed list of variables and risk factors of interest extracted from SEER.**

| **Variable** | **NAACCR Name** |
| --- | --- |
| Year | Year of diagnosis |
| Location = breast | CS schema v0204+ |
| First cancer per patient | Sequence number - central |
| Breast cancer HR/HER2 status | Breast subtype (2010+) |
|  | CS site-specific factor 1 / estrogen receptor assay |
|  | CS site-specific factor 2 / progesterone receptor assay |
| Age | Age at diagnosis |
| Sex | Sex |
| Stage | Derived AJCC-7 Stage group |
| Distant metastases | CS Mets at diagnosis |
| Grade | CS site-specific Factor 7/ Nottingham or Bloom-Richardson score/Grade |
| Tumor size | CS tumor size |
| Number of positive ipsilateral axillary lymph nodes | Regional nodes positive |
|  | CS site-specific factor 3 / number of positive ipsilateral Level I-II axillary lymph nodes |
| Node status | CS lymph nodes |
| Survival months | Survival months |
| All-cause death | Vital status recode |
| Cancer specific death | SEER cause-specific death classification |
| Race/ethnicity | Origin recode NHIA (Hispanic, Non-Hispanic) |
|  | Race recode (White, Black, American Indian, Asian Pacific Islander) |
| Histology | Histologic type ICD-O-3 |

Abbreviations: AJCC-7, *American Joint Committee on Cancer Staging Manual*, 7^th^ edition; CS, collaborative stage; HER, human epidermal growth factor receptor; HR, hormone receptor; ICD-O-3, International Classification of Diseases for Oncology, 3^rd^ edition; NAACCR, The North American Association of Central Cancer Registries; NHIA, The NAACCR Hispanic/Latino Identification Algorithm; SEER, Surveillance, Epidemiology, and End Results Program.
