# Supplementary material for: Breast cancer-specific mortality in early breast cancer as defined by high-risk clinical and pathologic characteristics
Source: PLoS One. 2022 Feb 25;17(2):e0264637. doi: 10.1371/journal.pone.0264637 (PMC8880870; doi:10.1371/journal.pone.0264637)
Supplement: S2 Table — aChi-square p-value of mortality analytic cohort versus excluded cohort. bPer the American Joint Committee on Cancer Staging Manual, micrometastases were defined as tumor deposits larger than 0.2 mm but not larger than 2.0 mm in the largest dimension. Cases in which at least 1 micrometastasis is detected, but no metastases larger than 2 mm are detected, regardless of number involved are classified as pN1mi or pN1mi(sn). cIn these analyses, node positive was exclusive of the N1mi subgroups. Please refer to Materials and Methods section, Data Source subsection for detailed information regarding nodal status classification. dOther combines histologic subtypes with <1% of patients which included: phyllodes tumor, Paget disease, inflammatory adenocarcinoma, medullary adenocarcinoma, mucin-producing adenocarcinoma, tubular adenocarcinoma, adenocarcinoma not otherwise specified, epidermoid carcinoma, papillary adenocarcinoma, unspecified carcinoma, other specific carcinoma, unspecified, and other specific types. Abbreviations: HER2, human epidermal growth factor receptor 2; HR, hormone receptor; mi, microinvasive carcinoma; N1, node status; NOS, not otherwise specified; TNBC, triple negative breast cancer. (DOCX) [file pone.0264637.s004.docx]

**S2 Table. Demographic and clinical characteristics of the overall population of interest from SEER who met initial inclusion criteria, the mortality analytic cohort, and those excluded from the mortality analytic cohort.**

| **Variable** | **Overall Population of Interest**  **N=342149** | **Mortality Analytic Cohort n=228031** | **Excluded from Mortality Analytic Cohort**  **n=114118** | **p-value^a^** |
| --- | --- | --- | --- | --- |
| **Sex, n (%)** |  |  |  | <0.0001 |
| Male | 2560 (0.75) | 1591 (0.70) | 969 (0.85) |  |
| Female | 339589 (99.25) | 226440 (99.30) | 113149 (99.15) |  |
| **Age group, n (%)** |  |  |  | 0.0001 |
| 18-29 | 1719 (0.50) | 1279 (0.56) | 440 (0.39) |  |
| 30-39 | 13873 (4.05) | 10507 (4.61) | 3366 (2.95) |  |
| 40-49 | 52613 (15.38) | 39996 (17.54) | 12617 (11.06) |  |
| 50-59 | 81431 (23.80) | 59077 (25.91) | 22354 (19.59) |  |
| 60-69 | 93495 (27.33) | 63753 (27.96) | 29742 (26.06) |  |
| 70-79 | 63226 (18.48) | 38545 (16.90) | 24681 (21.63) |  |
| 80-89 | 30826 (9.01) | 13829 (6.06) | 16997 (14.89) |  |
| 90+ | 4966 (1.45) | 1045 (0.46) | 3921 (3.44) |  |
| **Race/ethnicity** |  |  |  | <0.0001 |
| Spanish-Hispanic-Latino | 37237 (10.88) | 26342 (11.55) | 10895 (9.55) |  |
| Non-Hispanic White | 236409 (69.10) | 155197 (68.06) | 81212 (71.16) |  |
| Non-Hispanic Black | 36319 (10.61) | 23707 (10.40) | 12612 (11.05) |  |
| Non-Hispanic American Indian/ Alaska Native | 1891 (0.55) | 1309 (0.57) | 582 (0.51) |  |
| Non-Hispanic Asian or Pacific Islander | 28716 (8.39) | 20418 (8.95) | 8298 (7.27) |  |
| Non-Hispanic unknown | 1577 (0.46) | 1058 (0.46) | 519 (0.45) |  |
| **Breast subtype** |  |  |  | <0.0001 |
| HR+, HER2+ | 32682 (9.55) | 22332 (9.79) | 10350 (9.07) |  |
| HR-, HER2+ | 13696 (4.00) | 8822 (3.87) | 4874 (4.27) |  |
| HR+, HER2- | 238222 (69.63) | 163259 (71.60) | 74963 (65.69) |  |
| TNBC | 35761 (10.45) | 23614 (10.36) | 12147 (10.64) |  |
| HR+, HER2 unknown | 12206 (3.57) | 6397 (2.81) | 5809 (5.09) |  |
| Other | 9582 (2.80) | 3607 (1.58) | 5975 (5.24) |  |
| **Stage** |  |  |  | <0.0001 |
| Stage I NOS/IA | 176512 (51.59) | 114108 (50.04) | 62404 (54.68) |  |
| Stage IB | 7969 (2.33) | 6150 (2.70) | 1819 (1.59) |  |
| Stage IIA | 78229 (22.86) | 53207 (23.33) | 25022 (21.93) |  |
| Stage IIB | 39596 (11.57) | 28358 (12.44) | 11238 (9.85) |  |
| Stage III NOS/IIIA | 22642 (6.62) | 16287 (7.14) | 6355 (5.57) |  |
| Stage IIIB-C | 17201 (5.03) | 9921 (4.35) | 7280 (6.38) |  |
| **Nodal status** |  |  |  | <0.0001 |
| Node negative | 242467 (72.59) | 155541 (68.72) | 86926 (80.74) |  |
| Micrometastasis 1–3 positive ipsilateral axillary nodes | 13514 (4.05) | 10704 (4.73) | 2810 (2.61) |  |
| Micrometastasis ≥4 positive ipsilateral axillary nodes^b^ | 406 (0.12) | 306 (0.14) | 100 (0.09) |  |
| Node positive 1–3 positive ipsilateral axillary nodes^c^ | 54814 (16.41) | 42168 (18.63) | 12646 (11.75) |  |
| Node positive ≥4 positive ipsilateral axillary nodes^c^ | 22803 (6.83) | 17625 (7.79) | 5178 (4.81) |  |
| **Laterality** |  |  |  | <0.0001 |
| Left | 168492 (49.25) | 112555 (49.36) | 55937 (49.02) |  |
| Right | 173507 (50.71) | 115449 (50.63) | 58058 (50.88) |  |
| Other | 150 (0.04) | 27 (0.01) | 123 (0.11) |  |
| **Histology** |  |  |  | <0.0001 |
| Other adenocarcinomas | 4170 (1.22) | 2602 (1.14) | 1568 (1.37) |  |
| Mucinous adenocarcinoma | 6809 (1.99) | 4124 (1.81) | 2685 (2.35) |  |
| Infiltrating duct carcinoma | 254575 (74.40) | 173870 (76.25) | 80705 (70.72) |  |
| Lobular carcinoma, NOS | 31870 (9.31) | 20013 (8.78) | 11857 (10.39) |  |
| Infiltrating duct mixed/infiltrating lobular mixed | 32116 (9.39) | 21719 (9.52) | 10397 (9.11) |  |
| Other^d^ | 12609 (0.04) | 5703 (0.03) | 6906 (0.06) |  |

^a^Chi-square p-value of mortality analytic cohort versus excluded cohort.

^b^Per the *American Joint Committee on Cancer Staging Manual*, micrometastases were defined as tumor deposits larger than 0.2 mm but not larger than 2.0 mm in the largest dimension. Cases in which at least 1 micrometastasis is detected, but no metastases larger than 2 mm are detected, regardless of number involved are classified as pN1mi or pN1mi(sn).

^c^In these analyses, node positive was exclusive of the N1mi subgroups. Please refer to Materials and Methods section, Data Source subsection for detailed information regarding nodal status classification.

^d^Other combines histologic subtypes with <1% of patients which included: phyllodes tumor, Paget disease, inflammatory adenocarcinoma, medullary adenocarcinoma, mucin-producing adenocarcinoma, tubular adenocarcinoma, adenocarcinoma not otherwise specified, epidermoid carcinoma, papillary adenocarcinoma, unspecified carcinoma, other specific carcinoma, unspecified, and other specific types.

Abbreviations: HER2, human epidermal growth factor receptor 2; HR, hormone receptor; mi, microinvasive carcinoma; N1, node status; NOS, not otherwise specified; TNBC, triple negative breast cancer.
