# Supplementary material for: Breast cancer-specific mortality in early breast cancer as defined by high-risk clinical and pathologic characteristics
Source: PLoS One. 2022 Feb 25;17(2):e0264637. doi: 10.1371/journal.pone.0264637 (PMC8880870; doi:10.1371/journal.pone.0264637)
Supplement: S3 Table — aNot statistically significant; indicates p≥0.05 in either the model building and/or model validation halves. bSignificant at p<0.05 level in the model building and model validation halves, and p<0.0001 in the overall model. cPer the American Joint Committee on Cancer Staging Manual, micrometastases were defined as tumor deposits larger than 0.2 mm but not larger than 2.0 mm in the largest dimension. Cases in which at least 1 micrometastasis is detected, but no metastases larger than 2 mm are detected, regardless of number involved are classified as pN1mi or pN1mi(sn). dIn these analyses, node positive was exclusive of the N1mi subgroups. Please refer to Materials and Methods section, Data Source subsection for detailed information regarding nodal status classification. eOther combines other adenocarcinomas, mucinous adenocarcinoma, and histologic subtypes with <1% of patients which included: phyllodes tumor, Paget disease, inflammatory adenocarcinoma, medullary adenocarcinoma, mucin-producing adenocarcinoma, tubular adenocarcinoma, adenocarcinoma not otherwise specified, epidermoid carcinoma, papillary adenocarcinoma, unspecified carcinoma, other specific carcinoma, unspecified, and other specific types. Abbreviations: CI, confidence interval; HER2, human epidermal growth factor receptor 2; HR, hormone receptor; mi, microinvasive carcinoma; N1, node status; NOS, not otherwise specified; TNBC, triple negative breast cancer. (DOCX) [file pone.0264637.s005.docx]

**S3 Table. Multivariable Cox proportional hazards regression results for breast cancer-specific mortality.**

|  | **Overall Multivariable Model** |
| --- | --- |
|  | **Hazard Ratio (95% CI)** |
| **Subtype (reference, HR+, HER2-)** | |
| HR+, HER2 unknown | 0.97 (0.84,1.12)^a^ |
| HR+, HER2+ | 0.83 (0.77, 0.90)^b^ |
| HR-, HER2+ | 1.27 (1.16, 1.38)^b^ |
| Other | 1.56 (1.37, 1.79)^b^ |
| TNBC | 2.64 (2.51, 2.78)^b^ |
| **Age group (reference, 18-29)** | |
| 30-39 | 1.15 (0.92, 1.43)^a^ |
| 40-49 | 0.92 (0.74, 1.13)^a^ |
| 50-59 | 1.04 (0.84, 1.29)^a^ |
| 60-69 | 1.16 (0.94, 1.43)^a^ |
| 70-79 | 1.73 (1.40, 2.14)^b^ |
| 80-89 | 3.02 (2.43, 3.74)^b^ |
| 90+ | 5.14 (3.99, 6.62)^b^ |
| **Race/ethnicity (reference, Non-Hispanic White)** | |
| Spanish-Hispanic-Latino | 1.09 (1.03, 1.17)^a^ |
| Non-Hispanic Black | 1.41 (1.34, 1.49)^b^ |
| Non-Hispanic American Indian/Alaska Native | 1.18 (0.93, 1.51)^a^ |
| Non-Hispanic Asian or Pacific Islander | 0.69 (0.63, 0.76)^b^ |
| Non-Hispanic unknown | 0.19 (0.09, 0.42)^b^ |
| **Stage (reference, Stage I NOS/IA)** | |
| Stage IB | 1.30 (1.04, 1.63)^a^ |
| Stage IIA | 1.51 (1.37, 1.67)^b^ |
| Stage IIB | 1.75 (1.51, 2.02)^b^ |
| Stage III NOS/IIIA | 2.05 (1.74, 2.40)^b^ |
| Stage IIIB-C | 3.77 (3.21, 4.42)^b^ |
| **Nodal status (reference, node negative)** | |
| Micrometastasis 1–3 positive ipsilateral axillary nodes | 1.45 (1.26, 1.67)^b^ |
| Micrometastasis ≥4 positive ipsilateral axillary nodes^c^ | 3.86 (2.89, 5.14)^b^ |
| Node positive 1–3 positive ipsilateral axillary nodes^d^ | 1.79 (1.65, 1.95)^b^ |
| Node positive ≥4 positive ipsilateral axillary nodes^d^ | 2.62 (2.35, 2.91)^b^ |
| **Grade (reference, Grade 1)** | |
| Grade 2 | 1.41 (1.30, 1.52)^2^ |
| Grade 3 | 2.61 (2.41, 2.83)^2^ |
| **Tumor size (reference, <1 cm)** | |
| ≥1 cm to <2 cm | 1.46 (1.31, 1.62)^2^ |
| ≥2 cm to <3 cm | 1.83 (1.62, 2.07)^2^ |
| ≥3 cm to <4 cm | 2.47 (2.17, 2.82)^2^ |
| ≥4 cm to <5 cm | 3.01 (2.63, 3.44)^2^ |
| ≥5 cm | 3.61 (3.15, 4.12)^2^ |
| **Sex (reference, female)** | |
| Male | 1.19 (0.96, 1.46)^1^ |
| **Histology (reference, infiltrating duct/lobular mixed)** | |
| Lobular carcinoma, NOS | 1.00 (0.90, 1.11)^1^ |
| Medullary adenocarcinoma | 0.38 (0.23, 0.61)^2^ |
| Other^e^ | 1.18 (1.09, 1.28)^2^ |

^a^Not statistically significant; indicates p≥0.05 in either the model building and/or model validation halves.

^b^Significant at p<0.05 level in the model building and model validation halves, and p<0.0001 in the overall model.

^c^Per the *American Joint Committee on Cancer Staging Manual*, micrometastases were defined as tumor deposits larger than 0.2 mm but not larger than 2.0 mm in the largest dimension. Cases in which at least 1 micrometastasis is detected, but no metastases larger than 2 mm are detected, regardless of number involved are classified as pN1mi or pN1mi(sn).

^d^In these analyses, node positive was exclusive of the N1mi subgroups. Please refer to Materials and Methods section, Data Source subsection for detailed information regarding nodal status classification.

^e^Other combines other adenocarcinomas, mucinous adenocarcinoma, and histologic subtypes with <1% of patients which included: phyllodes tumor, Paget disease, inflammatory adenocarcinoma, medullary adenocarcinoma, mucin-producing adenocarcinoma, tubular adenocarcinoma, adenocarcinoma not otherwise specified, epidermoid carcinoma, papillary adenocarcinoma, unspecified carcinoma, other specific carcinoma, unspecified, and other specific types.

Abbreviations: CI, confidence interval; HER2, human epidermal growth factor receptor 2; HR, hormone receptor; mi, microinvasive carcinoma; N1, node status; NOS, not otherwise specified; TNBC, triple negative breast cancer.
