# Supplementary material for: Breast cancer-specific mortality in early breast cancer as defined by high-risk clinical and pathologic characteristics
Source: PLoS One. 2022 Feb 25;17(2):e0264637. doi: 10.1371/journal.pone.0264637 (PMC8880870; doi:10.1371/journal.pone.0264637)
Supplement: S4 Table — aPer the American Joint Committee on Cancer Staging Manual, micrometastases were defined as tumor deposits larger than 0.2 mm but not larger than 2.0 mm in the largest dimension. Cases in which at least 1 micrometastasis is detected, but no metastases larger than 2.0 mm are detected, regardless of number involved are classified as pN1mi or pN1mi(sn). bIn these analyses, node positive was exclusive of the N1mi subgroups. Please refer to Materials and Methods section, Data Source subsection for detailed information regarding nodal status classification. cOther combines other adenocarcinomas, mucinous adenocarcinoma, and histologic subtypes with <1% of patients which included: phyllodes tumor, Paget disease, inflammatory adenocarcinoma, medullary adenocarcinoma, mucin-producing adenocarcinoma, tubular adenocarcinoma, adenocarcinoma not otherwise specified, epidermoid carcinoma, papillary adenocarcinoma, unspecified carcinoma, other specific carcinoma, unspecified, and other specific types. Abbreviations: HER2, human epidermal growth factor receptor 2; HR, hormone receptor; mi, microinvasive carcinoma; N1, node status; NE, not estimable; NOS, not otherwise specified; TNBC, triple negative breast cancer. (DOCX) [file pone.0264637.s006.docx]

**S4 Table. Hazard ratio and sample-size adjusted chi-square values for breast cancer-specific mortality risk factors by HR, HER2 subtype.**

| **Hazard Ratio**  **Sample-size Adjusted Chi-square** | **HR+, HER2-** | **TNBC** | **HR+, HER2+** | **HR-, HER2+** |
| --- | --- | --- | --- | --- |
| **Risk Factors Presented in Figure 2 and S1 Figure** | | | | |
| **Race/ethnicity (reference, Non-Hispanic White)** | | | | |
| Spanish-Hispanic-Latino | 1.13  0.00004 | 1.02  0.000005 | 1.03  0.000007 | 1.21  0.0002 |
| Non-Hispanic Black | 1.50  0.0006 | 1.21  0.0007 | 1.41  0.001 | 1.71  0.001 |
| Non-Hispanic American Indian/Alaska Native | 1.25  0.00001 | 0.87  0.00001 | 1.28  0.00003 | 1.71  0.0001 |
| Non-Hispanic Asian or Pacific Islander | 0.66  0.0002 | 0.74  0.0005 | 0.68  0.0008 | 0.66  0.0003 |
| Non-Hispanic unknown | 0.19  0.00005 | 0.11  0.0002 | NE | 0.40  0.00004 |
| **Nodal status (reference, node negative)** | | | | |
| Micrometastasis 1–3 positive ipsilateral axillary nodes | 1.18  0.00002 | 1.91  0.001 | 1.58  0.0002 | 1.44  0.00008 |
| Micrometastasis ≥4 positive ipsilateral axillary nodes^a^ | 2.78  0.0001 | 4.56  0.001 | 7.70  0.002 | 4.11  0.0003 |
| Node positive 1–3 positive ipsilateral axillary nodes^b^ | 1.58  0.0003 | 2.04  0.004 | 1.98  0.002 | 2.00  0.0009 |
| Node positive ≥4 positive ipsilateral axillary nodes^b^ | 2.05  0.0005 | 3.46  0.007 | 2.12  0.001 | 3.74  0.002 |
| **Histologic grade (reference, Grade 1)** | | | | |
| Grade 2 | 1.47  0.0004 | 0.85  0.0001 | 1.20  0.00007 | 1.19  0.00005 |
| Grade 3 | 3.61  0.004 | 1.05  0.00001 | 1.31  0.0002 | 1.44  0.0002 |
| **Tumor size (reference, <1 cm)** |  |  |  |  |
| ≥1 cm to 2 cm | 1.38  0.0001 | 1.53  0.0006 | 1.87  0.001 | 1.95  0.0005 |
| ≥2 cm to 3 cm | 1.63  0.0002 | 1.84  0.001 | 2.31  0.001 | 3.16  0.001 |
| ≥3 cm to 4 cm | 2.31  0.0005 | 2.48  0.002 | 2.73  0.002 | 3.50  0.001 |
| ≥4 cm to 5 cm | 2.78  0.0007 | 2.91  0.003 | 3.22  0.002 | 4.67  0.002 |
| ≥5 cm | 3.07  0.0008 | 4.07  0.005 | 3.66  0.003 | 5.38  0.002 |
| **Additional Risk Factors Not Presented in S1 Figure** | | | | |
| **Sex (reference, female)** |  |  |  |  |
| **Male** | 1.05  0.000001 | 3.58  0.0003 | 2.31  0.00008 | 1.43  0.00007 |
| **Age group (reference, 18-29)** | | | | |
| 30-39 | 1.00  0.000000 | 1.21  0.00005 | 2.75  0.0002 | 0.97  0.000000 |
| 40-49 | 0.71  0.00002 | 1.09  0.00001 | 2.64  0.0002 | 0.81  0.00002 |
| 50-59 | 0.87  0.000004 | 1.12  0.00002 | 2.78  0.0002 | 1.03  0.000000 |
| 60-69 | 0.97  0.000000 | 1.13  0.00002 | 3.42  0.0003 | 1.34  0.00004 |
| 70-79 | 1.52  0.00004 | 1.52  0.0003 | 5.14  0.0006 | 2.09  0.0002 |
| 80-89 | 2.64  0.0002 | 2.20  0.0009 | 12.32  0.001 | 4.66  0.001 |
| 90+ | 3.67  0.0003 | 4.05  0.002 | 28.28  0.002 | 13.28  0.002 |
| **Stage (reference, Stage I NOS/IA)** | | | | |
| Stage IB | 1.65  0.00007 | 1.25  0.00004 | 1.01  0.000000 | 0.72  0.00002 |
| Stage IIA | 1.69  0.0003 | 1.54  0.0009 | 1.10  0.00003 | 0.95  0.000004 |
| Stage IIB | 2.16  0.0003 | 1.66  0.0007 | 1.09  0.00001 | 0.93  0.000004 |
| Stage III NOS/IIIA | 2.80  0.0005 | 1.66  0.0005 | 1.62  0.0003 | 1.07  0.000003 |
| Stage IIIB-C | 5.05  0.001 | 3.09  0.003 | 3.27  0.002 | 1.64  0.0001 |
| **Histology (reference, infiltrating duct carcinoma)** | | | | |
| Lobular carcinoma, NOS | 1.09  0.00001 | 0.83  0.00005 | 1.09  0.000003 | 0.90  0.00001 |
| Medullary adenocarcinoma | 0.39  0.00002 | 0.25  0.0007 | 1.72  0.0001 | 1.53  0.00001 |
| Other^c^ | 1.17  0.0001 | 1.04  0.00001 | 1.37  0.0002 | 1.29  0.0001 |

^a^Per the *American Joint Committee on Cancer Staging Manual*, micrometastases were defined as tumor deposits larger than 0.2 mm but not larger than 2.0 mm in the largest dimension. Cases in which at least 1 micrometastasis is detected, but no metastases larger than 2.0 mm are detected, regardless of number involved are classified as pN1mi or pN1mi(sn).

^b^In these analyses, node positive was exclusive of the N1mi subgroups. Please refer to Materials and Methods section, Data Source subsection for detailed information regarding nodal status classification.

^c^Other combines other adenocarcinomas, mucinous adenocarcinoma, and histologic subtypes with <1% of patients which included: phyllodes tumor, Paget disease, inflammatory adenocarcinoma, medullary adenocarcinoma, mucin-producing adenocarcinoma, tubular adenocarcinoma, adenocarcinoma not otherwise specified, epidermoid carcinoma, papillary adenocarcinoma, unspecified carcinoma, other specific carcinoma, unspecified, and other specific types.

Abbreviations: HER2, human epidermal growth factor receptor 2; HR, hormone receptor; mi, microinvasive carcinoma; N1, node status; NE, not estimable; NOS, not otherwise specified; TNBC, triple negative breast cancer.
